# A retinoic acid-Rfx6 cascade modulates Wnt/Bmp-dependent gut tube patterning: insight into the molecular basis of Mitchell–Riley Syndrome

**DOI:** 10.1242/dev.205552

**Published:** 2026-09-29

**Authors:** Scott A. Rankin, Jacek Biesiada, Kyle W. McCracken, Riya K. Patel, Mehek Rana, David M. Luedeke, Daniel O. Kechele, James M. Wells, Aaron M. Zorn

**Affiliations:** Division of Developmental Biology, Center for Stem Cell and Organoid Medicine (CuSTOM), Cincinnati Children's Hospital and the University of Cincinnati, College of Medicine, Cincinnati, OH 45229, USA

**Keywords:** Retinoic acid, RFX6, Endoderm, Wnt, Bmp, Foregut, *Xenopus*, PSCs, Mitchell–Riley Syndrome

## Abstract

Multiple signaling pathways and transcription factors (TFs) establish organ domains in the developing gastrointestinal tract. How these are integrated into spatial-temporal networks to regulate organogenesis and how disruptions to those networks lead to congenital syndromes remain poorly understood. Using human pluripotent stem cell cultures and *Xenopus* embryos, we demonstrate that retinoic acid (RA) from the lateral plate mesoderm directly activates expression of the TF *rfx6* in posterior foregut endoderm. Rfx6 subsequently promotes posterior foregut identity while suppressing Wnt-dependent hindgut and Bmp-dependent pharyngeal fates through direct and indirect mechanisms. Rfx6 can directly activate the expression of several key foregut TFs (*onecut1* and *pdx1*) and Wnt antagonists (*sfrp2/5*) while indirectly restricting expression of Wnt and Bmp ligands. Rfx6 also directly suppresses transcription of the Wnt-dependent hindgut TF *cdx2* and the Bmp-dependent pharyngeal TFs *nkx2-5* and *nkx2-6*. Thus, Rfx6 acts at multiple levels to integrate RA, Wnt and Bmp activity into a network with lineage-promoting TFs to control gut tube patterning. These results provide insight into the molecular basis of Mitchell–Riley Syndrome congenital anomalies, which are caused by *RFX6* mutations.

## Teaser

**Highlighted Article:** An RA-Rfx6 cascade promotes foregut identity by restricting Wnt-dependent hindgut and BMP-dependent pharyngeal fates.

## INTRODUCTION

Development of the digestive and respiratory systems from the embryonic endoderm requires temporal and spatial coordination of retinoic acid (RA), Wnt and Bmp signals. These signals pattern the gut tube along the anterior-posterior (A-P) and dorsal-ventral (D-V) axes into foregut, midgut and hindgut domains that are progressively refined into organ primordia by lineage-promoting transcription factors (TFs) (Bayha et al., 2009; Zorn and Wells, 2009). How these different growth factor pathways and TFs are mechanistically integrated into regulatory networks to control organogenesis remains poorly understood. Addressing this is crucial because genetic mutations in signaling pathways and TFs, or their disruptions by teratogenic environmental exposure, can result in congenital anomalies. Pioneering work dating back to the 1940s by Josef Warkany and colleagues at Cincinnati Children's Hospital demonstrated that maternal vitamin A-deficiency in rats led to widespread organ defects in offspring (Wilson et al., 1953). We now know that vitamin A is the precursor to RA and that disruptions in RA signaling in both humans and animal models results in pleiotropic congenital defects in the cardiac, pulmonary and digestive systems (Leon et al., 2023; Shannon et al., 2017; Lipscomb et al., 2006; Wang et al., 2006; Sandell et al., 2012; Guertin et al., 2022; Teletin et al., 2023).

RA plays a key role in gut tube patterning, having temporal and dose-dependent effects (Kelly and Drysdale, 2015; Green et al., 2011; Zhang et al., 2013; Loh et al., 2014; McCracken et al., 2014; Rankin et al., 2018; Kearns et al., 2023; Cipriano et al., 2024). After gastrulation, as the gut tube elongates, RA is produced by splanchnic lateral plate mesoderm that lies adjacent to the foregut – high levels of RA promote posterior foregut and lower levels of RA promote anterior foregut pharyngeal fates (Green et al., 2011; Kearns et al., 2023; Cipriano et al., 2024). The RA-responsive transcriptional programs and patterning mechanisms that define anterior foregut versus posterior foregut identity are poorly understood. RA also plays roles in D-V foregut patterning. The RA-synthesizing enzyme Aldh1a2 is more robustly expressed in dorsal splanchnic mesoderm, and studies have shown that the dorsal pancreatic bud is more sensitive to RA than the ventral bud (Molotkov et al., 2005; Martín et al., 2005; Chen et al., 2004); however, ventral hepatobiliary development does require RA (Guertin et al., 2022; Koike et al., 2019).

Wnt and Bmp also have dynamic roles during gut tube patterning and foregut organogenesis. During the early somite stages of development, when RA is patterning the foregut, Wnt and Bmp ligands expressed in posterior lateral plate mesoderm promote hindgut intestinal fates (McLin et al., 2007; Spence et al., 2011; Stevens et al., 2017; Tsai et al., 2017; Munera et al., 2017; Garriock et al., 2020). The posteriorizing activity of these pathways is limited by secreted Wnt antagonists (Sfrps and Frzbs) and BMP antagonists (Noggin and Chordin) in the anterior mesendoderm and early foregut, where they are required to permit foregut specification (Li et al., 2008; Green et al., 2011). Later in development, after foregut, midgut and hindgut regional domains are established, the impact of Wnt and Bmp signaling changes. Bmp promotes pharyngeal (Kurmann et al., 2015; Lovely et al., 2016; Li et al., 2019; Kearns et al., 2023) and liver (Wandzioch and Zaret, 2009; Loh et al., 2014; Palaria et al., 2018) fates and combinatorial Wnt/Bmp induces lung (Goss et al., 2009; Domyan et al., 2011). While we have some understanding of how RA, Wnt and Bmp individually impact gut tube patterning, it is unclear how the activity of these pathways is integrated.

In this study, we identified a conserved RA-responsive transcriptional target, the winged-helix TF Rfx6, and demonstrate it is crucial for regulating multiple signaling inputs during gut tube patterning. Rfx6 is expressed in embryonic foregut and midgut endoderm (Smith et al., 2010; Pearl et al., 2011) and recessive mutations in human *RFX6* cause Mitchell–Riley Syndrome (OMIM #615710), a congenital disorder with variably penetrant multi-organ defects that include pancreas hypoplasia, annular pancreas, gall bladder agenesis, extrahepatic biliary atresia, intestinal atresia and intestinal malrotation (Mitchell et al., 2004; Smith et al., 2010). While Rfx6 function specifically in pancreatic, small intestine and endocrine cell development has been well studied (Smith et al., 2010; Soyer et al., 2010; Pearl et al., 2011; Piccand et al., 2014, 2019; Gehart et al., 2019; Trott et al., 2020; Ibrahim et al., 2024; Sanchez et al., 2024), its role in foregut patterning was unclear. We show that Rfx6 is an RA-induced TF that promotes posterior foregut identity while suppressing Wnt-dependent hindgut and Bmp-dependent pharyngeal fates. By identifying target genes and investigating Rfx6-responsive enhancers, we determine that Rfx6 acts as both an activator and repressor and functionally integrates the activities of RA, Wnt and Bmp into a network with multiple lineage-promoting TFs to control gut tube patterning. These results provide insight into the molecular basis of human Mitchell–Riley Syndrome.

## RESULTS

### *RFX6* is part of a conserved transcriptional program induced by RA in posterior foregut

The transcriptional program induced by RA in the gut tube endoderm that promotes posterior foregut fate is not well understood (Fig. 1A). To identify RA-regulated genes that are evolutionarily conserved during foregut development, we performed bulk RNA-sequencing on human pluripotent stem cell (hPSC)-derived foregut endoderm cultures and micro-dissected *Xenopus laevis* foregut endoderm explants treated with or without exogenous RA (Fig. 1B,C). In hPSC cultures, we employed a well-established differentiation protocol that mimics fetal foregut development (McCracken et al., 2014; Rankin et al., 2016). For *Xenopus* embryos, we dissected the foregut tissue at Nieuwkoop and Faber (NF) stage 15 (NF15) and removed the lateral plate mesoderm, the endogenous source of RA. The dose and timing of RA exposure for each species were chosen to optimally promote posterior foregut fate based on multiple published studies (Green et al., 2011; Loh et al., 2014; McCracken et al., 2014; Rankin et al., 2021; Kearns et al., 2023; Cipriano et al., 2024). A comparable developmental stage when foregut identity is established was assayed: day 6 of hPSC differentiation and NF25 in *Xenopus* (Fig. 1B,C).

**Fig. 1.**
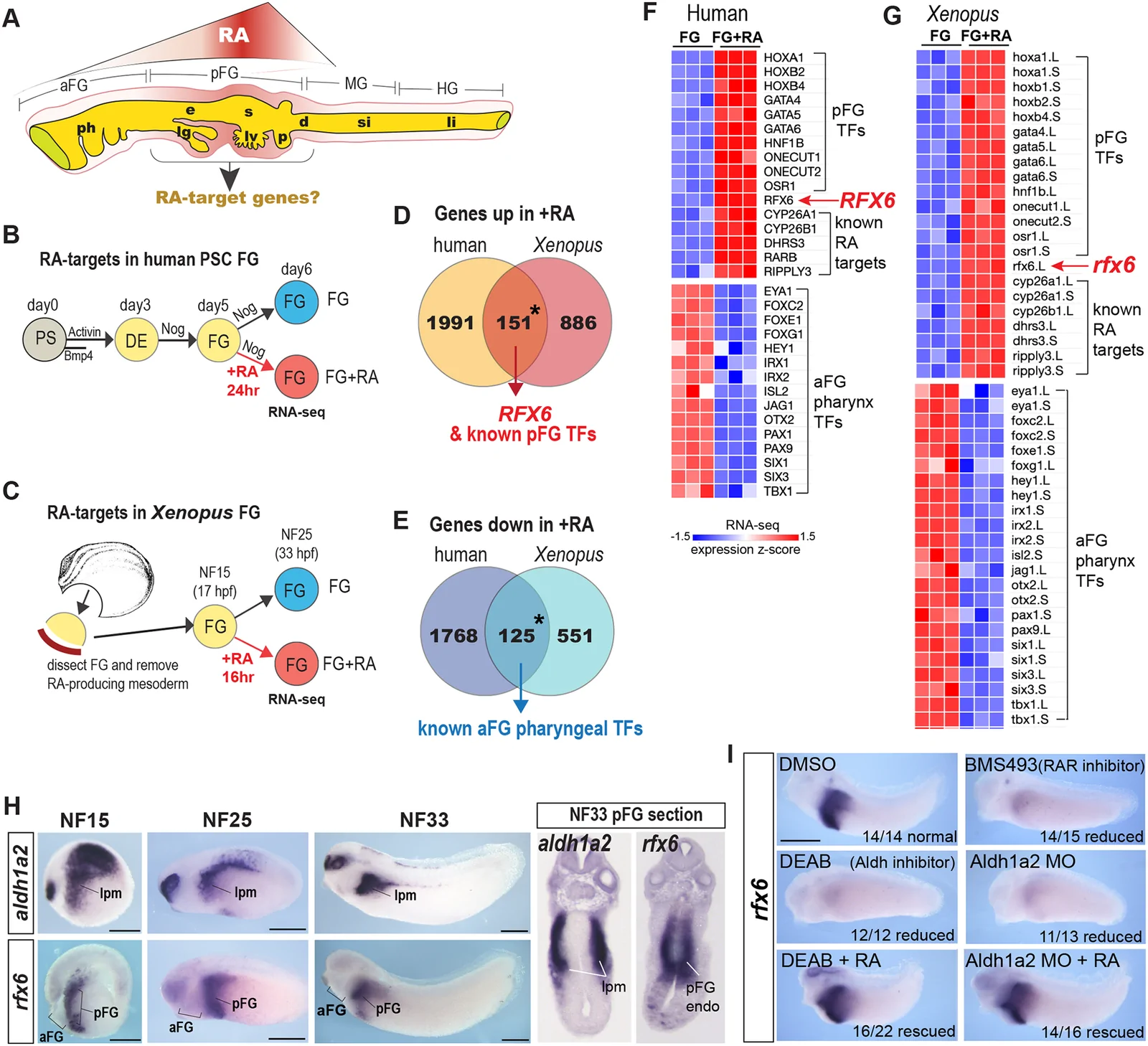
***Rfx6* is an RA-activated gene in human and *Xenopus* foregut endoderm.** (A) Schematic of the goal to identify RA-target genes that pattern the posterior foregut. aFG, anterior foregut; pFG, posterior foregut; MG, midgut; HG, hindgut; ph, pharynx; e, esophagus; lg: lung; s, stomach; lv, liver; p, pancreas; d, duodenum; si, small intestine; li, large intestine. (B,C) Experimental design using hPSCs (B) and *Xenopus* (C) embryos to identify RA-regulated genes in foregut (FG) endoderm. hpf, h post fertilization. (D,E) Overlap of day 6 hPSC and NF25 *Xenopus* RA-responsive differentially expressed mRNAs+RA; Log2 fold-change≥0.5, FDR<0.05. Asterisk indicates hypergeometric test *P*<5×10^−5^. (F,G) Heat maps showing the expression of curated RA targets and TFs in day 6 hPSC (F) and NF25 *Xenopus* (G) foregut endoderm±RA. Each column is a biological replicate. (H) *In situ* hybridization of *X. laevis* embryos at the indicated stages, comparing expression of the RA synthesizing enzyme *aldh1a2* to *rfx6*. (I) *In situ* hybridization of *rfx6* in *X. tropicalis* embryos at NF33, demonstrating that endogenous RA signaling is required for *rfx6* expression. Scale bars: 500 μm.

Differential expression analysis identified 2142 upregulated and 1893 downregulated genes in hPSC-derived foregut exposed to RA from day 5-6 (24 h) and 1037 upregulated and 676 downregulated genes in *Xenopus* foregut endoderm treated with RA from NF15-25 (16 h) (Fig. 1D,E; Fig. S1A,B; Table S1). Intersecting the human and *Xenopus* RA-responsive transcriptomes revealed 151 genes upregulated and 125 genes downregulated by RA in both species (Fig. 1D,E). The common upregulated genes included well-known direct RA targets such as *HOXA1*, *HOXB2*, *CYP26A1*, *DHRS3*, *RARB* and *RIPPLY3*, as well as posterior foregut genes including *GATA4/5/6*, *HNF1B*, and *OSR1*, reported in previous studies to be activated by RA (Fig. 1F,G) (Green et al., 2011; Loh et al., 2014; McCracken et al., 2014; Gere-Becker et al., 2018). Consistent with published work in hPSCs, multiple anterior foregut pharyngeal TFs, including *FOXE1*, *PAX1*, *PAX9*, *SIX1* and *TBX1*, were suppressed by RA in both species (Fig. 1F,G) (Green et al., 2011; Kearns et al., 2023; Cipriano et al., 2024).

Intriguingly, amongst the RA-activated genes in both humans and frogs was the winged helix TF *RFX6*, a known regulator of pancreatic and gut development with DNA variants known to cause Mitchell–Riley Syndrome (Smith et al., 2010; Pearl et al., 2011). We followed up on Rfx6 for several reasons: (1) transcriptional control of *rfx6* expression is poorly understood; (2) its function during gut tube patterning is not well characterized; and (3) the organ malformations observed in Mitchell–Riley Syndrome are reminiscent of the RA loss-of-function phenotypes in animal models, suggesting that Rfx6 might be a key effector of RA-dependent gut tube patterning.

*In situ* hybridization of *X. laevis* embryos from NF15 through NF33, when the gut tube is being patterned, confirmed that *rfx6* is expressed in posterior foregut endoderm immediately adjacent to lateral plate mesoderm that expresses the RA-synthesizing enzyme *aldh1a2* (Fig. 1H). We tested if *rfx6* expression required endogenous RA signaling using multiple approaches in both *X. laevis* and *Xenopus tropicalis* (*X. tropicalis*). Treatment of *X. laevis* or *X. tropicalis* embryos with the pharmacological agent DEAB, which blocks retinaldehyde dehydrogenase activity and hence RA production, or BMS493, a pan RA-receptor (RAR) antagonist, abolished or strongly reduced *rfx6* expression (Fig. 1I; Fig. S1C,D), as did injection of a well-characterized antisense morpholino targeting Aldh1a2 (*aldh1a2*-MO; Strate et al., 2009; Rankin et al., 2021) (Fig. 1I). Treating DEAB or *aldh1a2*-MO embryos with exogenous RA rescued *rfx6* expression (Fig. 1I). We conclude that *rfx6* is a conserved RA-induced gene during gut tube patterning.

### RA directly activates *RFX6* transcription via an evolutionarily conserved enhancer

We next tested whether RA can directly induce *rfx6* transcription by treating *X. laevis* foregut endoderm explants with a well-established method in which translation is blocked by cycloheximide (CHX) treatment (Fig. 2A). If a gene is still transcribed in response to an experimental treatment when translation is blocked, it must be directly activated (Kolm and Sive, 1995; Sinner et al., 2004). In contrast, if CHX blocks the expression of a target gene in response to the experimental treatment, then it is considered an indirect target activated by an intermediate factor. Indeed, RA was sufficient to activate *rfx6* transcription in the presence of CHX, demonstrating direct activation (Fig. 2B). In contrast, RA activation of the foregut TF *onecut1* was blocked by CHX, demonstrating indirect activation, while expression of the pan endodermal TF *foxa1* was unaffected (Fig. 2B).

**Fig. 2.**
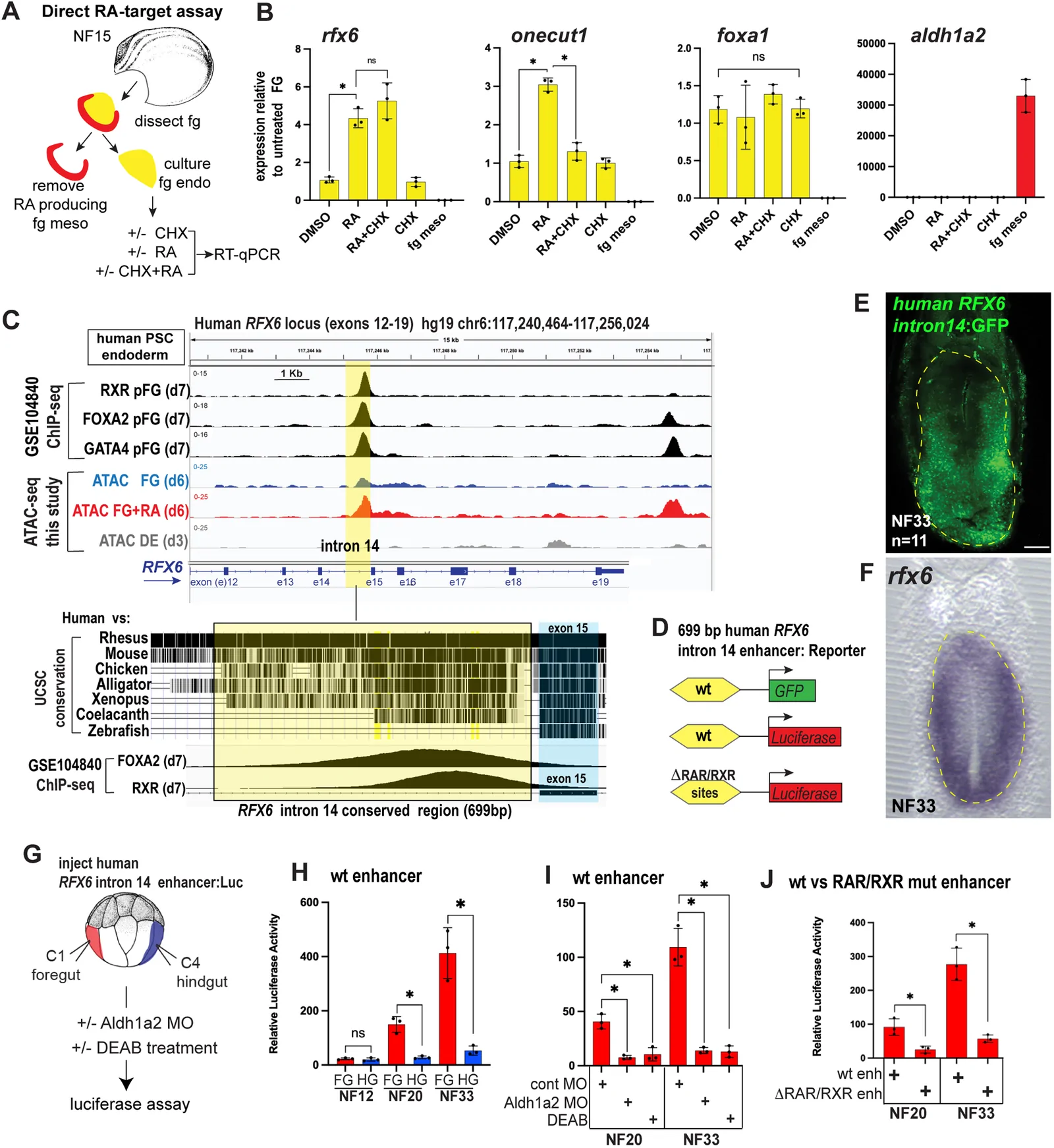
**RA directly activates *RFX6* transcription via an evolutionarily conserved intronic enhancer.** (A) Direct RA target assay in *X. laevis*. Foregut (FG) endoderm was dissected from stage NF15 embryos and pretreated with cycloheximide (CHX) or DMSO for 2 h before culture in CHX+RA for 6 h. (B) RT-qPCR analysis showing mean relative expression±standard deviation. *P<0.05, parametric two-tailed paired *t*-test. Each black dot is a pool of *n*=4 explants. (C) IGV genome browser view of the human *RFX6* locus showing the evolutionarily conserved 14th intron enhancer (yellow). Published ChIP-seq tracks of RXR, FOXA2 and GATA4 from day 7 hPSC posterior foregut (pFG) (Vinckier et al., 2020, GSE104840, in which RA treatment is days 5-7). ATAC-seq generated in this study from hPSC day 3 DE or day 6 FG±RA. (D) Schematic of 699 bp human RFX6 14th intron reporter constructs. wt, wild type. (E,F) The human *RFX6* intron 14 enhancer drives pFG GFP expression in F0 transient transgenics (E), similar to endogenous *rfx6* (F). Sections through the foregut. Dashed lines outline endoderm. *n=*11 refers to the number of embryos observed with GFP expression in the pFG obtained from two transgenesis experiments. (G) Experimental design of *Xenopus* luciferase assays. (H-J) Mean relative luciferase activity±standard deviation; *P<0.05, parametric two-tailed paired *t*-test. (H) The *RFX6* intronic reporter is active in the FG, but not hindgut (HG), at NF20 and NF33. (I) Endogenous RA signaling is required for enhancer activity. Injection of *aldh1a2*-MO or treatment with DEAB to inhibit RA activity. (J) Mutation of the identified RAR/RXR DNA-binding motifs (see Fig. S2D) reduced reporter activity. Scale bar: 100 μm (for E,F).

The DNA cis-regulatory elements governing RA-dependent *RFX6* expression in the embryonic gut tube have not been characterized, although recent studies have identified some enhancers that may regulate *RFX6* during pancreas development (Kaplan et al., 2024). To help identify candidate RA-responsive *RFX6* enhancers, we performed ATAC-seq on hPSC-derived day 3 definitive endoderm (DE) and foregut endoderm cultures treated with or without the 24-h RA dose from day 5-6. This revealed a region in the 14th intron of human *RFX6* that showed increased chromatin accessibility in response to RA (Fig. 2C), mirroring the RA-dependent transcriptional pattern of *RFX6* (Figs S1 and S2B). Examination of published ChIP-seq data from hPSC-derived posterior foregut/pancreatic endoderm cultures (Vinckier et al., 2020) showed that this accessible region in intron 14 was bound by the retinoid X receptor RXR (the required binding partner for all nuclear RARs), as well as by the endoderm pioneering TFs FOXA2 and GATA4 (Fig. 2C). This RXR-bound region in intron 14 was the only RXR peak±200 kb from the *RFX6* transcription start site (Fig. S2B; Vinckier et al., 2020), it displayed evolutionary conservation across vertebrate species (Fig. 2C; Fig. S2C), and the orthologous region in the *X. laevis rfx6* intron 14 was bound by p300 (a mark of active enhancers) in *Xenopus* foregut tissue (Fig. S2A; Stevens et al., 2017). Motif analysis of the RXR-bound human *RFX6* intron 14 DNA sequence using CisBP (Weirauch et al., 2014) revealed the presence of multiple predicted RAR/RXR DNA-binding motifs, as well as several FOXA1/2 and GATA4/6 motifs (Fig. 2C; Fig. S2D,E; Table S2).

To functionally test this putative enhancer, we generated reporter constructs containing the human 699 bp *RFX6* intron 14 sequence driving GFP or luciferase (Fig. 2D) and assayed their activity in *X. laevis*. The human *RFX6* intron 14 sequence was sufficient to drive transgenic GFP expression in the *Xenopus* posterior foregut endoderm, similar to endogenous *rfx6* (Fig. 2E,F). To quantitatively assess the spatial-temporal activity of this human enhancer and interrogate the requirement of the identified RAR/RXR motifs, we performed targeted micro-injection (Fig. 2G) of the wild type (WT) or of an RAR/RXR motif mutant (ΔRAR) luciferase reporter, in which we scrambled the RAR/RXR motifs (Fig. S2D,E). The WT human enhancer drove robust luciferase activity in the foregut but not the hindgut at NF20 and NF33, and not earlier at gastrula NF12 (Fig. 2H), consistent with the endogenous *rfx6* expression pattern. Disruption of endogenous RA signaling via DEAB treatment or *aldh1a2*-MO injection significantly reduced WT enhancer activity (Fig. 2I), as did mutation of the RAR/RXR motifs (Fig. 2J). Collectively, these data suggest that RA signaling directly promotes the expression of *RFX6* in posterior foregut endoderm, which involves an evolutionarily conserved and RA-responsive enhancer located in the 14th intron.

### RA and Rfx6 promote posterior foregut while suppressing hindgut and pharyngeal fates

Previous studies of RA loss-of-function (LOF) (Lipscomb et al., 2006; Rankin et al., 2016) and Rfx6 LOF (Smith et al., 2010; Pearl et al., 2011) have independently reported similar hypoplastic foregut phenotypes; however, the molecular basis of both RA- and Rfx6-deficient foregut organogenesis defects remains poorly understood. We confirmed the strikingly similar RA and Rfx6 LOF phenotypes, directly comparing the gut tubes from sibling *X. laevis* embryos with RA LOF (treated with pan RAR antagonist BMS493 from NF15-33) or Rfx6 LOF (*rfx6* morpholino oligo injection; Pearl et al., 2011), which include severe hypoplasia of the pancreas, duodenum and stomach (Fig. 3A,B), akin to Mitchell–Riley Syndrome congenital anomalies. Injection of mRNA encoding a dexamethasone (DEX) inducible human *GR-RFX6* construct and DEX-induction starting at NF15 was sufficient to rescue the organ hypoplasia defects in *rfx6*-MO embryos (Fig. 3B), confirming the specificity of this previously validated translation-blocking morpholino oligo (Pearl et al., 2011; Sanchez et al., 2024). The GR-RFX6 construct has the hormone-binding domain of the glucocorticoid receptor (GR) fused in frame to human RXF6 (Sanchez et al., 2024; Kolm and Sive, 1995).

**Fig. 3.**
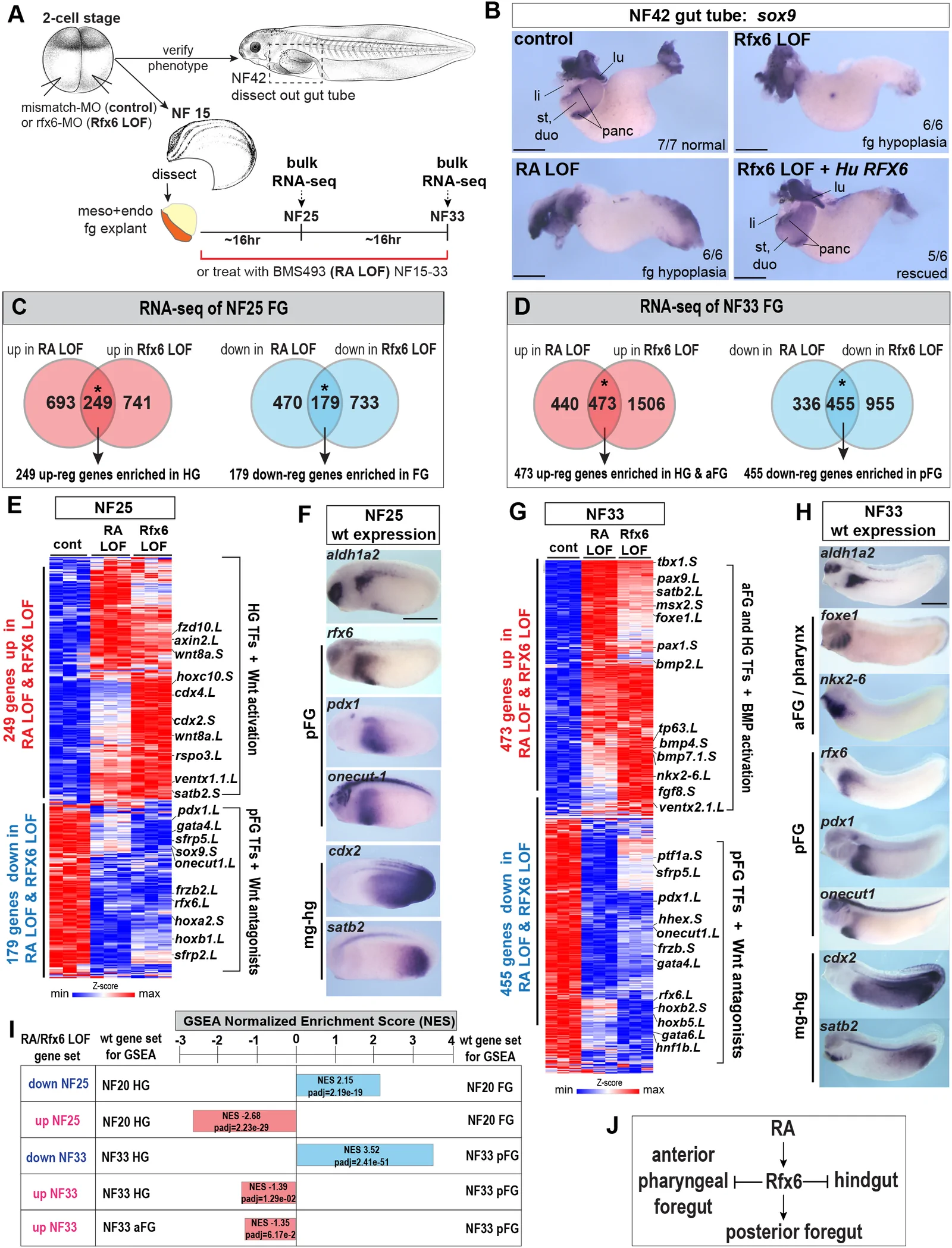
**RA and Rfx6 promote posterior foregut and suppress pharyngeal and hindgut fates.** (A) Experimental design for comparing RA-loss-of-function (LOF) and Rfx6-LOF phenotypes and transcriptomes. *Xenopus* embryo illustrations from Zahn et al. (2022). (B) *Sox9 in situ* hybridization of dissected gut tubes from sibling stage NF42 embryos, confirming similar foregut hypoplasia in RA-LOF and Rfx6-LOF embryos. duo, duodenum; li, liver; lu, lung; panc, pancreas; st, stomach. Lower right numbers indicate the numbers of embryos with the shown phenotype. (C,D) Venn overlap of differentially expressed genes (Log2 fold-change≥1.5, FDR<0.05) reveals common down- or upregulated genes in response to RA LOF and Rfx6 LOF; asterisk indicates hypergeometric test *P*<7×10^−117^. (E,G) Heat map of genes commonly upregulated or downregulated in RA and Rfx6 LOF at NF25 (E) and NF33 (G). (F,H) *In situ* hybridization of control wild-type (WT) *X. laevis* embryos at NF25 (F) and NF33 (H) showing the normal expression patterns in anterior foregut (aFG), posterior foregut (pFG), midgut (mg) and hindgut (hg). (I) Gene set enrichment analysis (GSEA) of RA/Rfx6 co-regulated genes at NF25 and NF33 shows that RA/Rfx6 LOF downregulated genes are enriched for pFG genes whereas RA/Rfx6 LOF upregulated genes are enriched for HG and aFG/pharyngeal genes. (J) Model of RA and Rfx6-regulated patterning. Scale bars: 500 μm.

To directly compare the RA-dependent transcriptional program to that regulated by Rxf6, we performed bulk RNA-seq on foregut explants (containing both endoderm and Aldh1a2^+^ mesoderm) micro-dissected from control, RA LOF (BMS493-treated NF15-33) or Rfx6 LOF (*rfx6*-MO) sibling *Xenopus* embryos at multiple stages. Explants were dissected at NF15, assayed at NF25 during RA-mediated patterning and at NF33 when organ lineages are induced (Horb and Slack, 2001; Shifley et al., 2012; Rankin et al., 2018) (Fig. 3A). Differential expression analysis revealed that the RA-dependent and Rfx6-dependent transcriptomes shared significant overlap at both NF25 and NF33 (Fig. 3C,D; Table S3). At NF25, there were 249 upregulated and 179 downregulated genes common to both RA-LOF and Rfx6-LOF (Fig. 3C,E), while at NF33, there were 473 upregulated and 455 downregulated genes in common (Fig. 3D,G). Strikingly, at NF33, Rfx6 regulated ∼50% of the same genes as RA, suggesting that Rfx6 is a key mediator of RA patterning. Interestingly, the RA/Rfx6 co-regulated genes included many TFs known to be regionally expressed along the A-P axis (Fig. 3E,G), indicating altered endoderm patterning. For example, foregut TFs including *pdx1*, *hnf1b* and anterior *hox1-5* were downregulated, while hindgut TFs such as *cdx2*, *satb2* and posterior *hox9-11* were upregulated in both RA-LOF and Rfx6-LOF foregut tissue at both NF25 and NF33 (Fig. 3E-H; Fig. S3J).

Gene set enrichment analysis (GSEA) confirmed an abnormal A-P pattern in the RA/Rfx6 LOF tissue at both stages (Fig. 3I). We compared the NF25 RA/Rfx6 co-regulated genes with the transcriptional profiles of previously published *X. laevis* foregut and hindgut tissues (Fig. S3A; Table S4) (Stevens et al., 2017). This confirmed that the 249 genes upregulated in RA/Rfx6 LOF at NF25 were normally enriched in the hindgut, whereas the 179 RA/Rfx6 downregulated genes were normally enriched in the foregut (Fig. 3I; Fig. S3C,D). For the later NF33 time point, we compared the RA/Rfx6 dysregulated genes to RNA-seq profiles we generated from control NF33 embryos micro-dissected into anterior foregut/pharyngeal, posterior foregut, or hindgut tissue (Fig. S3B; Table S4). The NF33 GSEA confirmed that the genes downregulated in RA/Rfx6 LOF were normally enriched in the posterior foregut, while the upregulated genes were enriched for genes normally expressed in the control hindgut and pharynx (Fig. 3I; Fig. S3E-G), confirming altered gut tube pattern.

The GSEA findings were corroborated by Gene Ontology (GO) analysis, with the RA/Rfx6 common downregulated genes being enriched for terms including A-P patterning, epithelium development, GI, heart, and respiratory system development (Fig. S3H,I). GO terms enriched in the RA/Rfx6 upregulated genes also included A-P patterning, pharyngeal development, and the skeletal system. Intriguingly, ‘canonical Wnt Signaling’ was a significant GO term in both the up- and downregulated gene sets at NF25. Indeed, the heatmap shows that canonical Wnt/β-catenin pathway genes and expression of Wnt-responsive hindgut TFs (*cdx2* and ventx family members) were upregulated in both RA LOF and Rfx6 LOF foregut tissue. In contrast, the NF25-downregulated genes included secreted Wnt antagonists (Fig. 3E,F). GO analyses of the RA/Rfx6 upregulated genes at NF33 revealed an enrichment for Bmp-signaling, consistent with increased expression of *bmp2*, *bmp4*, *bmp7* and Bmp-response gene *msx2* and pharyngeal TFs (*tbx1*, *pax1*, *pax9*, *foxe1*, *nkx2-6*) (Fig. 3G; Fig. S3I,J). Interestingly, these BMP-regulated and pharyngeal genes were already upregulated in the RA LOF foregut at NF25, but not until NF33 in the Rfx6 LOF (Fig. S4A,C). While a substantial number of genes were uniquely regulated by either RA or Rfx6, GO enrichment analysis suggested that, overall, these transcriptional programs are involved in similar biological processes, including cell migration, ECM organization, anterior-posterior pattern, and mesoderm lineage development (Fig. S4B,D).

Lastly, we compared the genes identified by this RA LOF analysis (Fig. 3) to the genes activated by RA treatment (Fig. 1). This identified genes where RA was necessary and sufficient for their expression, including *rfx6*, along with pFG TFs *gata4*, *hnf1b*, *pdx1*, *osr1*, *onecut1* and *hoxa/b/c/d 1-5*, and which also required Rfx6 (Fig. S5A; Table S5). Moreover, a group of pharyngeal genes that were suppressed by the addition of RA (Fig. 1) were indeed upregulated in the RA LOF and/or RFX6 LOF (Fig. S5B; Table S5). This analysis also identified some genes in LOF conditions that were mesodermal or non-cell-autonomously regulated through interactions with the mesoderm, a tissue that was not present in the Fig. 1 foregut endoderm RA treatment experiment.

Collectively, these data suggest that a temporally dynamic RA-Rfx6 regulatory cascade promotes posterior foregut identity and represses hindgut and pharyngeal fates, possibly by suppressing Wnt, Bmp and key TFs of the hindgut and pharyngeal lineages (Fig. 3J). RA suppresses both hindgut and pharyngeal markers at both NF25 and NF33, whereas Rfx6 only suppresses Wnt-dependent hindgut markers at NF25, and it is not until NF33 that it suppresses both hindgut and BMP-dependent pharyngeal markers.

### Rfx6 promotes foregut identity and suppresses Wnt/β-catenin-dependent hindgut fate

The transcriptomic analysis suggested that between NF15-25, RA and Rfx6 regulate gut tube patterning in part by restraining canonical Wnt/β-catenin signaling, which promotes hindgut fate (McLin et al., 2007; Stevens et al., 2017; Kjolby and Harland, 2017; Nakamura et al., 2016). A closer examination of the RNA-seq data showed that canonical Wnt pathway genes were upregulated in both the RA LOF and Rfx6 LOF foregut tissue including *wnt8a*, Wnt receptors (*fzd10*, *rspo3*) and Wnt-response genes (*axin2*) and several Wnt-dependent hindgut TFs (*cdx2*, ventx genes, *satb2* and *hox7-12*) (Fig. 4A; Table S3). In contrast, foregut TFs (*hox1-3*, *gata4*, *onecut1*, *pdx1*, *sox9*) and secreted Wnt antagonists (*sfrp5*, *sfrp2* and *frzb2*), which protect the foregut endoderm from posterizing Wnt signals (Li et al., 2008), were downregulated (Fig. 4A; Table S3).

**Fig. 4.**
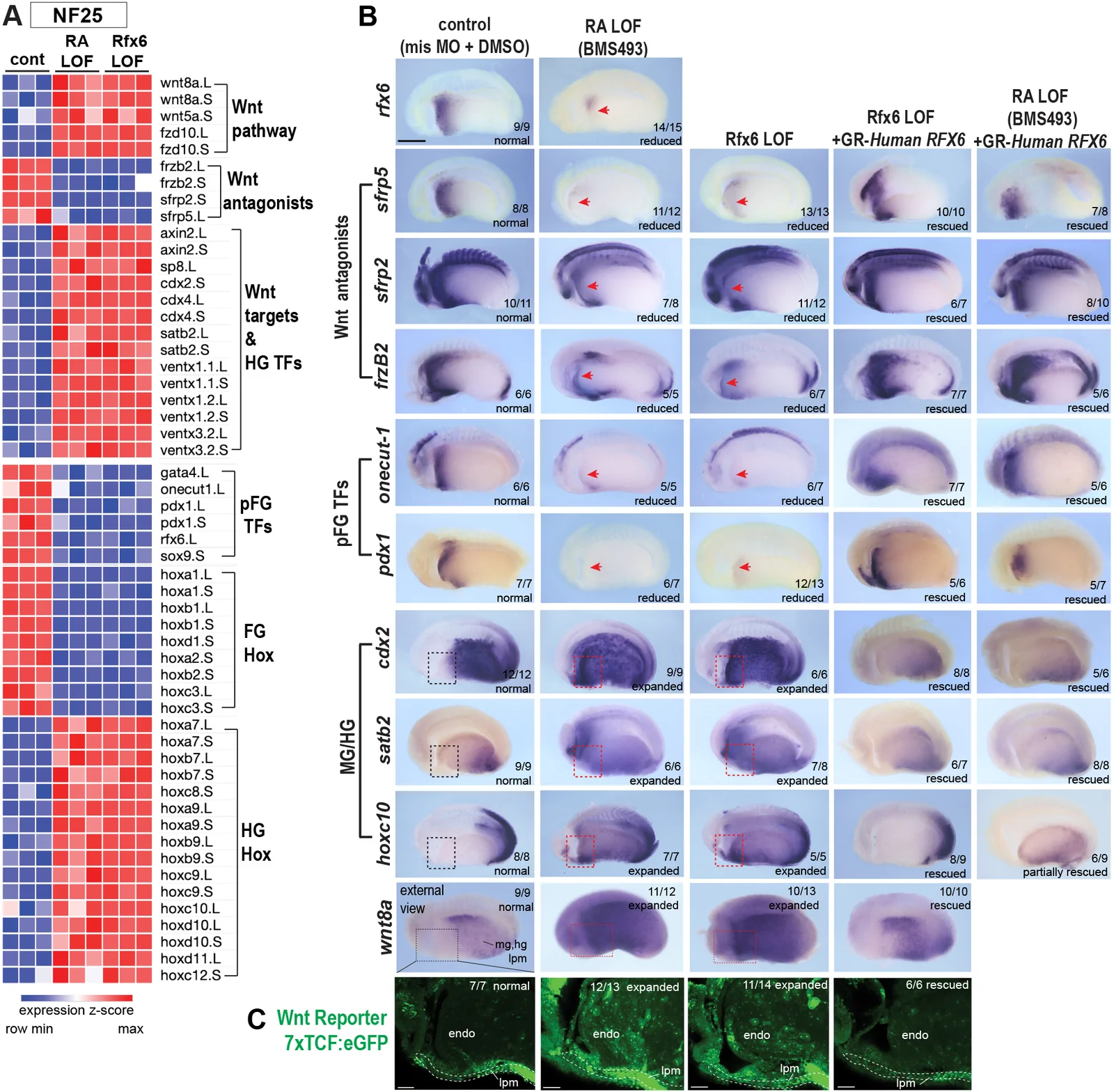
**RA and Rfx6 promote foregut identity and suppress Wnt-dependent hindgut fate.** (A) Curated heat map showing differentially expressed genes in RA and Rfx6 loss-of-function (LOF) at NF25. (B) *In situ* hybridization of NF25 *X. laevis* embryos showing spatial changes in A-P pattern due to RA and Rfx6 LOF. Internal views of bisected embryos are shown, except for *wnt8a*, which is an external view. Lower-right numbers indicate the number of embryos with the shown *in situ* hybridization phenotype. Red arrows highlight reduced expression, and red boxes ectopic expression. Continuous Dex treatment starting from NF15 in GR-Human RFX6 injected embryos. (C) GFP immunostaining of the NF25 foregut region from transgenic Wnt/β-catenin reporter *Tg(WntRE:dGFP) X. laevis* embryos. Scale bars: 100 µm. endo, endoderm; FG, foregut; HG, hindgut; lpm, lateral plate mesoderm; MG, midgut; pFG, posterior foregut. Scale bars: 500 µm (B); 100 µm (C).

We next performed *in situ* hybridization to examine how spatial expression domains changed in the RA/Rfx6 LOF. This confirmed that RA and Rfx6 are required for expression of *sfrp5*, *sfrp2*, *frzb2*, *onecut1* and *pdx1* in the posterior foregut and that RA and Rfx6 LOF embryos have expanded *cdx2*, *satb2* and *hoxc10* expression into the foregut domain (Fig. 4B). The canonical Wnt ligand *wnt8a*, which is usually restricted to the hindgut lateral late mesoderm, was expanded along the A-P axis into the foregut territory. This expansion, combined with the reduced expression of Wnt antagonists, suggested elevated canonical Wnt activity in the RA/Rfx6 LOF foregut. To directly examine canonical Wnt/β-catenin activity, we performed additional LOF experiments in a transgenic Wnt reporter line *Tg(WntRE:dGFP)* that expresses GFP under the control of a Wnt/Tcf/β-catenin responsive regulatory element (Tran et al., 2010). As predicted, ectopic Wnt-reporter GFP expression was observed in both the RA and Rfx6 LOF foregut endoderm and mesoderm (Fig. 4C).

We performed additional epistatic experiments to suppress the canonical Wnt pathway in *rfx6*-MO injected embryos and explants by treating with the Wnt/β-catenin inhibitor XAV939 or recombinant Dkk1, respectively (Fig. S6A). These reagents were sufficient to reduce the ectopic, elevated levels of *cdx2*, *hoxc10* and *wnt8a*; however, expression of foregut TFs *pdx1* and *onecut1* was not rescued by Wnt inhibition (Fig. S6B,C). This suggests a dual role of Rfx6 in promoting early posterior foregut fate: a role in activation or maintenance of foregut TFs that is separable from a role in maintaining low Wnt/β-catenin activity in the foregut and suppression of hindgut identity via regulation of secreted Wnt-antagonists and restriction of *wnt8* expression.

### Rfx6 directly activates transcription of secreted Wnt antagonists and foregut TFs while directly suppressing *cdx2*

Intriguingly, we found that GR-human RFX6 could rescue expression of Wnt antagonists (*sfrp5*, *sfrp2*, *frzb2*) and the TFs *pdx1* and *onecut1* in RA LOF embryos (Fig. 4B). To test whether Rfx6 can directly regulate the transcription of these patterning genes, we performed gain-of-function (GOF) experiments in *Xenopus* hindgut explants using the GR-human RFX6 construct and the translation inhibitor CHX, which was administered before and during DEX induction (Fig. 5A). We found that human GR-RFX6 activated expression of *sfrp5*, *sfrp2* and *onecut1* in the presence of CHX, similar to the known direct Rfx6 target *pdx1* (Sanchez et al., 2024). However, RFX6 induction of *frzb2* and *sox9* was blocked by CHX, indicating indirect activation (Fig. 5B). *Wnt8a*, *cdx2*, *satb2*, and *hoxc10* were all suppressed by RFX6 in the hindgut explants; amongst these, *wnt8a* (Fig. 5B) and *hoxc10* (Fig. 5B; Fig. S7A) displayed CHX-sensitive suppression, indicating that RFX6 indirectly repressed their transcription. In contrast, *cdx2* and *satb2* were still significantly reduced by GR-RFX6 in the presence of CHX (Fig. 5B; Fig. S7A), supporting a direct repressive function of RFX6. These data demonstrate that RFX6 can both directly activate and directly repress transcription of target genes (Fig. 5C).

**Fig. 5.**
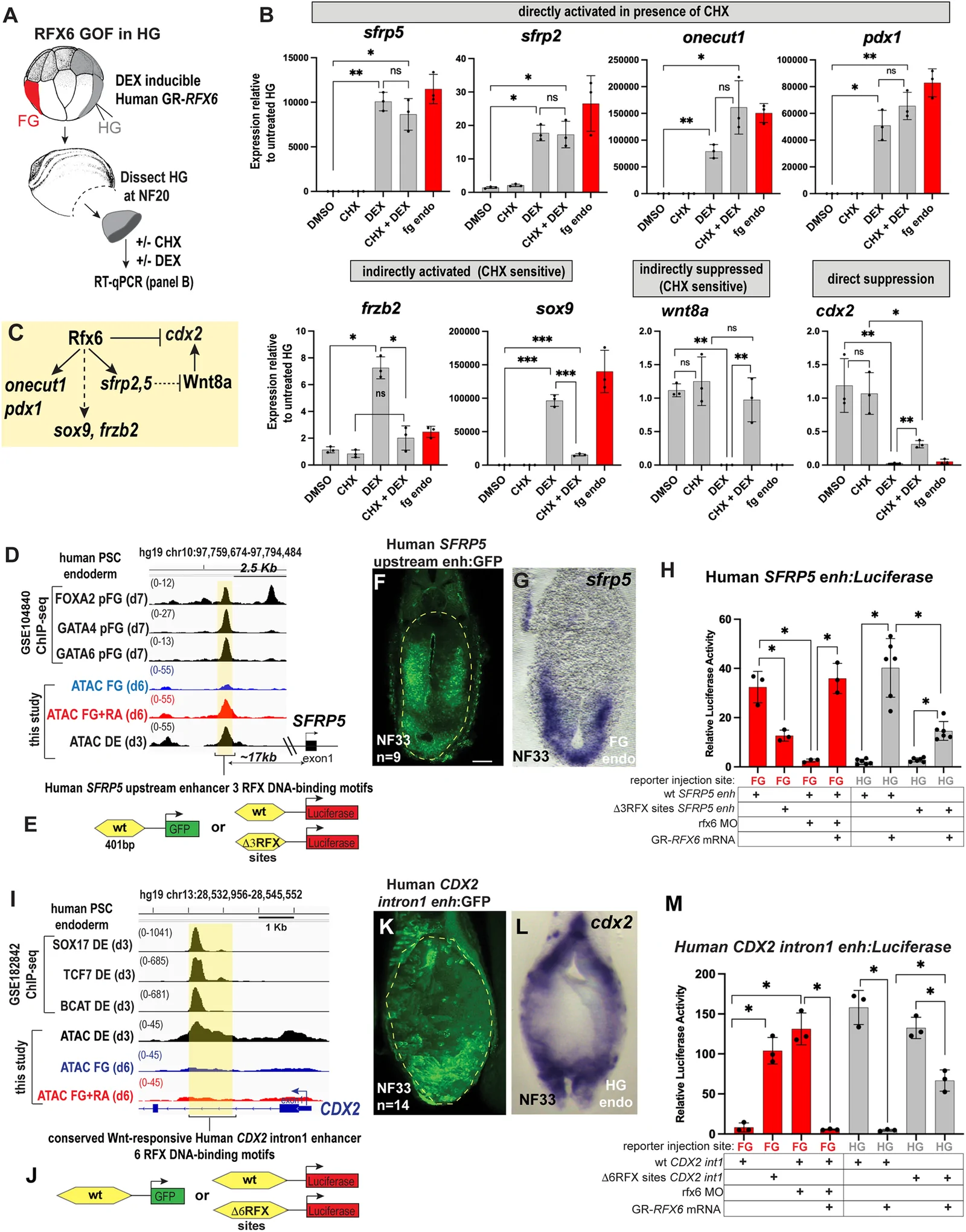
**Rfx6 can function as both a transcriptional activator of *sfrp5* and a repressor of *cdx2*.** (A) Direct RFX6 target assay in *X. laevis*. DEX inducible GR-Human *RFX6* mRNA was injected into the presumptive hindgut. Hindgut explants were dissected at NF20 and pretreated with CHX for 2 h before being treated with DEX, CHX+DEX, or DMSO vehicle control for 6 h. (B) RT-qPCR showing relative normalized gene expression±standard deviation. *P<0.05, **P<0.01, ***P<0.001, parametric two-tailed paired *t*-test. ns, not significant. Each black dot is a pool of *n*=4 explants. (C) Model of Rfx6 target gene regulation. Solid line, direct; dashed line, indirect. (D) IGV genome browser of candidate human *SFRP5* enhancer showing published ChIP-seq tracks (Vinckier et al., 2020; GSE104840) and ATAC-seq generated in this study. (E) Cartoons of reporter constructs. wt, wild type. (F,G) The 401 bp human *SFRP5* enhancer can drive GFP expression in transgenic *X. laevis* embryos, similar to endogenous *sfrp5* (G)*.* (H) In luciferase assays, the *SFRP5* enhancer can drive RFX6-dependent activity in the FG but not the HG. Mean relative luciferase activity±standard deviation. *P<0.05, parametric two-tailed paired *t*-test. (I) IGV genome browser of the human *CDX2* intron 1 enhancer, with published ChIP-seq (Mukherjee et al., 2022; GSE182842) and ATAC-seq tracks. (J) Cartoons of reporter constructs. (K,L) The *CDX2* enhancer can drive GFP expression in the HG of transgenic *X. laevis* embryos (K), similar to endogenous *cdx2* (L)*. n*=14 refers to the number of embryos observed with GFP expression in the hindgut obtained from two transgenic experiments. (M) *Xenopus* luciferase assays. The *CDX2* intron 1 enhancer is active in the HG but not the FG. Depletion of endogenous Rfx6 or mutation of the *RFX6* motifs results in ectopic FG expression. Mean relative luciferase activity±standard deviation; *P<0.05, parametric two-tailed paired *t*-test. Scale bar: 100 µm (for F,G,K,L).

### Characterization of the RFX6-responsive human *SFRP5* and *CDX2* enhancers

We next sought to identify the RFX6-responsive enhancers regulating *SFRP5* and *CDX2*, since they are key regulators of gut tube pattern. To our knowledge ChIP-grade antibodies that recognize endogenous Rfx6 are not available in any species, thus we were not able to directly assay Rfx6 chromatin binding. We therefore turned to our ATAC-seq of hPSC foregut endoderm, which revealed a candidate cis-regulatory element ∼−17 kb upstream of the *SFRP5* transcription start site that displayed increased chromatin accessibility upon RA treatment, when *SFRP5* is robustly expressed (Fig. 5D; Fig. S1, Fig. S7B; Table S1). Reanalysis of published ChIP-seq data showed that the pioneering endodermal TFs GATA4/6 and FOXA2 bind this −17 kb region in hPSC-derived DE and foregut (Vinckier et al., 2020; Heslop et al., 2021) (Fig. 5D). Moreover, DNA sequence motif analysis of this ∼400 bp region revealed three CisBP predicted RFX DNA-binding motifs (Fig. S7C). While the DNA sequence of the syntenic region is not conserved in *Xenopus*, the *X. laevis sfrp5* upstream region contains three p300-bound candidate enhancers with predicted RFX DNA-binding motifs upstream of the transcription start site (Fig. S7D), suggesting similar regulation.

We cloned the 401 bp human *SFRP5* −17 kb upstream candidate enhancer into reporter constructs and tested the activity in *Xenopus* (Fig. 5E-H). In transgenics, the *hSFRP5* −17 kb upstream enhancer was sufficient to drive GFP expression in foregut endoderm, similar to endogenous *sfrp5* (Fig. 5F,G) and, in quantitative luciferase assays, the human *SFRP5* enhancer drove robust activity in foregut but not hindgut (Fig. 5H). Depletion of endogenous Rfx6 (*rfx6*-MO) largely abolished luciferase activity, while GR*-*human RFX6 robustly activated the enhancer in hindgut tissue (Fig. 5H). Finally, mutation of the three predicted RFX motifs significantly reduced expression in the foregut and blunted the ability of human *RFX6* to induce reporter activity in the hindgut (Fig. 5H). These data suggest that RFX6 can directly activate *SFRP5* transcription in the posterior foregut via this distal upstream enhancer.

Since RFX6 could directly suppress *cdx2* expression in *Xenopus* (Fig. 5B), we next focused on a known evolutionarily conserved and Wnt/β-catenin activated *CDX2* intronic enhancer (Fig. 5I; Stevens et al., 2017; Gaunt et al., 2005; Mukherjee et al., 2022). This 1105 bp conserved *CDX2* enhancer contained six CisBP predicted RFX DNA-binding motifs in both human and *Xenopus* (Fig. S8A-D). Published ChIP-seq data show that in hPSC-derived DE this conserved intronic enhancer is bound by TCF7, β-CATENIN and SOX17 (Fig. 5I; Fig. S8A) (Mukherjee et al., 2022), and by β-catenin and p300 in *Xenopus* hindgut tissue (Fig. S8C) (Stevens et al., 2017). We generated reporter constructs (Fig. 5J) and observed that the human *CDX2* intron 1 enhancer was sufficient to drive GFP expression in the hindgut endoderm of transgenic *Xenopus* embryos, similar to endogenous *cdx2* (Fig. 5K,L). Luciferase reporter assays confirmed that the human *CDX2* intron1 enhancer drove robust activity in the hindgut, but not the foregut, and that depletion of endogenous Rfx6 (*rfx6*-MO) or mutation of the six predicted RFX motifs significantly upregulated enhancer activity in the foregut (Fig. 5M). These observations are consistent with Rfx6-dependent suppression. In further support of direct repression, GR*-*human RFX6 robustly repressed the WT *CDX2* enhancer activity in the hindgut and mutation of the six RFX DNA-binding sites blunted the suppression (Fig. 5M; Fig. S8E). Collectively, these data indicate that in the foregut RFX6 can function both as an activator and repressor, having locus-specific effects.

### RA/Rfx6 suppresses Bmp-dependent pharyngeal fate

Our transcriptome analysis of RA LOF and Rfx6 LOF foregut tissue at NF33 revealed an upregulation of multiple pharyngeal TFs (*pax1*, *pax9*, *foxe1*, *tbx1*, *nkx2-5*, *nkx2-6*), Bmp ligands (*bmp2*, *bmp4*, *bmp7*) and known direct Bmp target genes (*szl*, msx and ventx genes) (Fig. 6A). Immunostaining of RA and Rfx6 LOF embryos confirmed that phosphorylated Smad1/5/9, the nuclear effector of active Bmp signaling, was ectopically expanded into the posterior foregut (Fig. 6B). These results are consistent with previous work showing that Bmp signaling promotes pharyngeal fate (Kurmann et al., 2015; Lovely et al., 2016; Li et al., 2019) and that high RA represses it (Green et al., 2011; Kearns et al., 2023; Cipriano et al., 2024), which suggests that RFX6 mediates the effect of RA, in part by modulating Bmp signaling. In support of this hypothesis, re-examination of published transcriptomic data of induced pluripotent stem cell (iPSC)-derived pancreatic progenitors from human Mitchell–Riley syndrome patients (Trott et al., 2020) and comparison to our RA/Rfx6 dysregulated genes identified a conserved set of upregulated genes, including *BMP4*, *NKX2-5* and *NKX2-6* (Fig. S9A-C; Table S6). To further examine how Rxf6 might suppress Bmp-dependent pharyngeal fate, we focused on the TFs *nkx2-5* and *nkx2-6*, which in mice are redundantly required for pharyngeal endoderm development (Tanaka et al., 2001) and are both directly activated by BMP/Smad1 in the *Xenopus* foregut (Fig. S9F; Stevens et al., 2017; Sparrow et al., 2000).

**Fig. 6.**
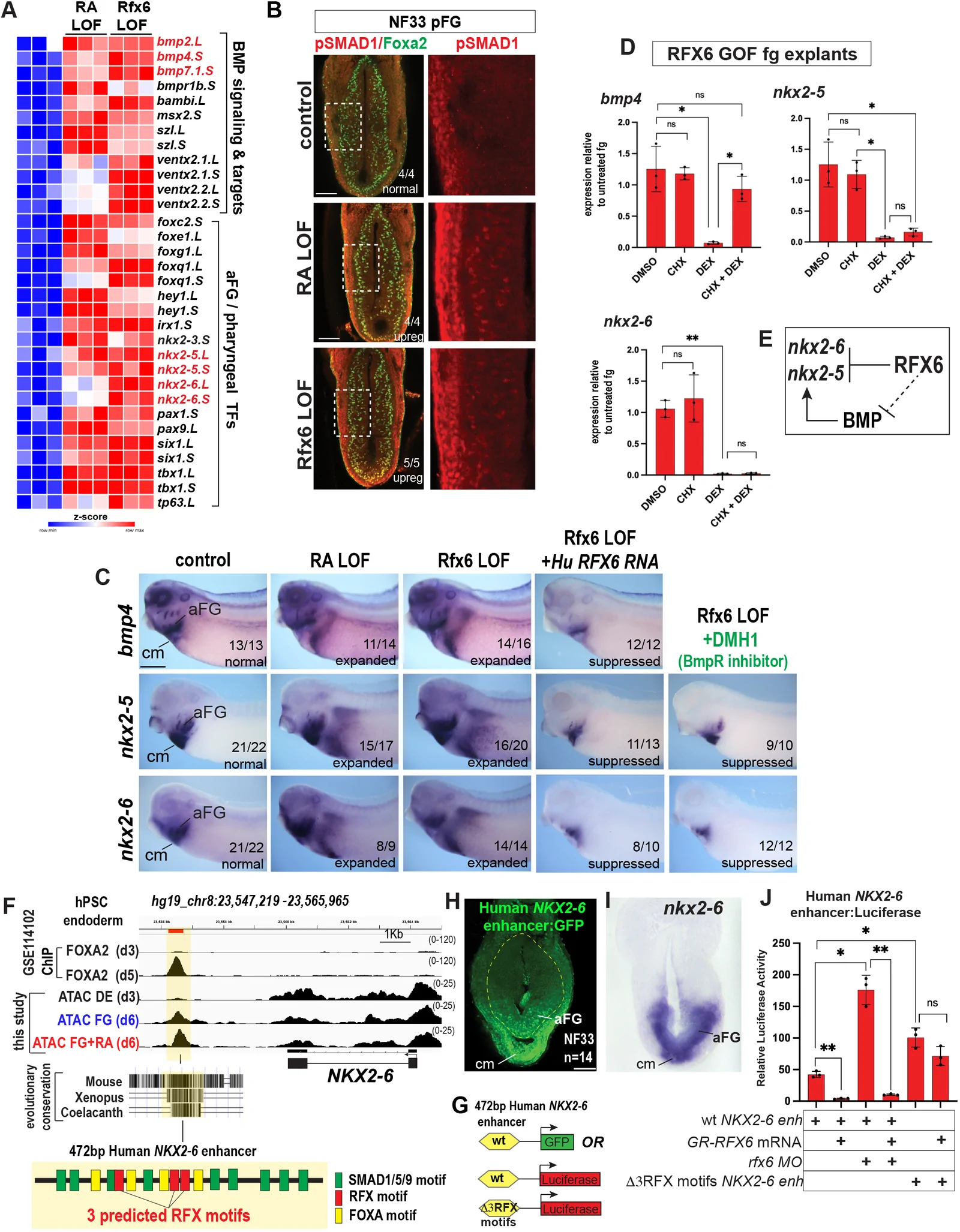
**RA and Rfx6 suppress Bmp-dependent pharyngeal endoderm fate and *nkx2-*6 expression.** (A) Curated heat map of differentially expressed Bmp pathway components, target genes and pharyngeal TFs. aFG, anterior foregut. (B) Immunostaining of NF33 control, RA loss-of-function (LOF) and Rfx6 LOF embryos shows ectopic phosphorylated Smad1/5/9 (pSMAD1; red) in the posterior foregut (pFG). Scale bars: 100 μm. (C) *In situ* hybridization of NF33 embryos showing ectopic *bmp4*, *nkx2-5* and *nkx2-6* caused by RA LOF and Rfx6 LOF, or rescue by 10 μM DMH1 treatment from NF25-NF33. (D) RT-qPCR analysis of RFX6 gain-of-function (GOF) foregut endoderm explants, in which RFX6 indirectly suppresses *bmp4* but directly suppresses *nkx2-6.* Foregut explants were dissected at NF20 and pretreated with CHX for 2 h before the addition of DEX, CHX+DEX or DMSO vehicle control for 6 h. Normalized relative expression±standard deviation; *P<0.05, **P<0.01, parametric two-tailed paired *t*-test. ns, not significant. Each black dot is a pool of *n*=4 explants. (E) Model indicating direct (solid line) and indirect (dashed line) suppressive activity of RFX6. (F) IGV genome browser of candidate human *NKX2-6* enhancer showing published FOXA2 ChIP-seq (Geusz et al., 2021; GSE114102; day 3 and day 5), ATAC-seq tracks from this study and evolutionary conservation. (G) Cartoons of reporter constructs. wt, wild type. (H,I) The *NKX2-6* enhancer can drive GFP expression in the pharyngeal aFG endoderm and cardiac mesoderm (cm) of transgenic *X. laevis* embryos (H), similar to endogenous *nkx2-6* (*in situ* hybridization in I). *n*=14 refers to numbers of embryos observed with GFP expression in aFG/cm obtained from two transgenesis experiments*.* (J) *Xenopus* luciferase assays at NF33. Foregut luciferase activity of the *NKX2-6* enhancer is suppressed by GR-RFX6 Dex treatment at NF20, and this suppression is dependent on RFX motifs. Mean relative luciferase activity±standard deviation; *P<0.05, **P<0.01, parametric two-tailed paired *t*-test. ns, not significant. Each black dot is a pool of *n*=5 embryos. Scale bars: 500 µm (B,C); 100 µm (for H,I).

*In situ* hybridization confirmed that *bmp4*, *nkx2-5* and *nkx2-6* were ectopically upregulated in the posterior foregut of RA LOF and Rfx6 LOF embryos (Fig. 6C). Treatment with the Bmp-receptor antagonist DMH1 eliminated the ectopic *nkx2-5* and *nkx2-6* expression in *rfx6*-MO embryos (Fig. 6C), confirming their BMP-dependent regulation and consistent with our hypothesis that Rfx6 suppresses pharyngeal fate in part via modulation of Bmp. To determine if Rfx6 could directly suppress expression of *bmp4*, *nkx2-5* and *nkx2-6*, we injected the GR*-*human RFX6 construct into the foregut, dissected foregut explants and induced RFX6 with or without the translation inhibitor CHX. While GR-RFX6 was sufficient to suppress all three genes, CHX treatment blocked the ability of RFX6 to suppress *bmp4*, indicating indirect suppression. In contrast, both *nkx2-5* and *nkx2-6* were still suppressed by RFX6 in the presence of CHX, demonstrating direct repression of their transcription (Fig. 6D,E).

### Characterization of an RFX6-suppressed human *NKX2-6* enhancer

We next sought to identify potential *NKX2-6* enhancers regulated by RFX6 based upon several criteria: (1) chromatin accessibility in hPSC-derived foregut and pharyngeal endoderm (Kearns et al., 2023; Cipriano et al., 2024); (2) FOXA2 binding in published ChIP-seq of hPSC foregut (Vinckier et al., 2020; Lee et al., 2019; Geusz et al., 2021); (3) evolutionary conservation and Smad1 binding in *Xenopus* foregut (Stevens et al., 2017); and (4) the predicted RFX DNA-binding motifs. This combinatorial approach identified a 472 bp evolutionarily conserved DNA element ∼8 kb downstream of the *NKX2-6* transcription start that contained three predicted RFX motifs and multiple predicted SMAD1/5/9 DNA-binding sites (Fig. 6F; Fig. S9E,G), consistent with BMP-dependent activation. The orthologous *Xenopus nkx2-6* region is bound by Smad1 and p300 in foregut tissue (Fig. S9F; Stevens et al., 2017).

We generated reporter constructs with the 472 bp human *NKX2-6* candidate downstream enhancer (Fig. 6G) and, in *Xenopus* transgenics, this sequence was sufficient to drive GFP expression in ventral pharyngeal endoderm and cardiac mesoderm at NF33, similar to endogenous *nkx2-6* (Fig. 6H,I). Depletion of endogenous *Xenopus* Rfx6 (*rfx6*-MO) or mutation of the three predicted RFX DNA-binding motifs resulted in elevated *NKX2-6* enhancer:luciferase activity in the foregut (Fig. 6J; Fig. S9G). In contrast, GR*-*human RFX6 suppressed reporter activity, and this suppression required the RFX motifs (Fig. 6J). We conclude that this conserved cis-regulatory element contributes to the pharyngeal expression of *NKX2-6* and is repressed by RFX6 in the posterior foregut. Collectively, these data suggest RFX6 can suppress Bmp-dependent pharyngeal fate in two ways: by indirectly and temporally restricting Bmp ligand expression as well as by direct repression of key pharyngeal TFs such as *nkx2-6* and *nkx2-5* (Fig. 6E).

## DISCUSSION

### Model of RA/Rfx6 dependent gut tube patterning

Multiple signaling pathways and TFs are involved in gut tube patterning, but how their activities are integrated into a coordinated regulatory network remains poorly understood. Here, we show that downstream of RA, the wing-helix transcription factor Rfx6 promotes posterior foregut identity by regulating expression of lineage-promoting TFs and restricting Wnt and Bmp ligand expression and pathway activity (Fig. 7). We demonstrate that RA from the foregut lateral plate mesoderm directly activates *rfx6* transcription in the adjacent posterior foregut endoderm. Rfx6 then promotes foregut and represses hindgut through several mechanisms: (1) It directly activates expression of several key posterior foregut TFs and secreted Wnt-antagonists in the endoderm. (2) These Wnt-antagonists act in a paracrine fashion to restrict canonical Wnt activity and *Wnt8* expression in the foregut. (3) Rfx6 directly suppresses transcription of the Wnt-responsive hindgut-specifying TF *cdx2.* Our temporal analysis indicates that RA suppresses both hindgut and pharyngeal genes starting at NF25, but that it is not until later in development at NF33 that Rfx6 indirectly restricts Bmp ligand expression and directly suppresses transcription of the Bmp-dependent pharyngeal-promoting TFs *nkx2-6* and *nkx2-5*. This work links RA, Wnt and Bmp into a network involving multiple lineage-specifying TFs and provides mechanistic insight into gut tube patterning.

**Fig. 7.**
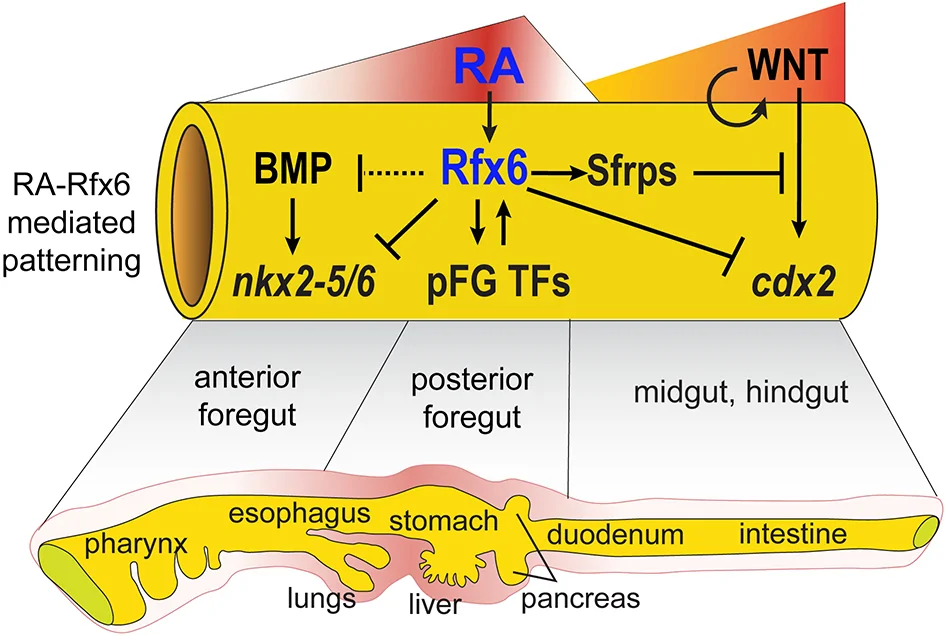
**Proposed model of RA and Rfx6-regulated gut tube patterning.** RA from the Aldh1a2^+^ lateral plate mesoderm adjacent to the posterior foregut endoderm directly activates expression of Rfx6. Rfx6 promotes posterior foregut identity while suppressing Wnt-dependent hindgut and BMP-dependent pharyngeal fates through direct and indirect mechanisms. Rfx6 can directly activate expression of several key foregut TFs (*onecut1* and *pdx1*) and secreted Wnt antagonists (*sfrp5*, *sfrp2*). Sfrps protect the foregut endoderm from posteriorizing Wnt signals. Rfx6 also directly suppresses transcription of the Wnt-dependent hindgut TF *cdx2* and the BMP-dependent pharyngeal TFs *nkx2-5* and *nkx2-6*. Thus, Rfx6 integrates the spatiotemporal activity of RA, Wnt and BMP pathways into a network with lineage-promoting TFs to control regional identity in the developing gut tube.

### A posterior foregut TF feed-forward loop downstream of RA

Downstream of RA, we hypothesize that RFX6 functions in positive feed-forward regulatory loops with other posterior foregut TFs. In mice, Pdx1, Onecut1 and Sox9 are known to promote each other's expression and suppress *Cdx2* at the foregut-midgut boundary (Shih et al., 2015), and we show that Rfx6 regulates these factors in *Xenopus*. Inspection of *ONECUT1*, *PDX1* and *HNF1B* ChIP-seq studies in human PSC foregut differentiation contexts reveals binding of these TFs to the *RFX6* proximal promoter and/or downstream regions during differentiation (Heller et al., 2021; Wang et al., 2018; Vinckier et al., 2020; El-Khairi et al., 2021), and *RFX6* levels are reduced in human HNF1B and PDX1 LOF hPSC studies (Zhu et al., 2016; El-Khairi et al., 2021; Wang et al., 2018). Thus, in our model we propose reciprocal arrows between RFX6 and posterior foregut TFs (Fig. 7), reflective of re-enforcing regulatory interactions that contribute to gut tube patterning.

### How does Rfx6 indirectly restrict Wnt and BMP ligand expression?

During *Xenopus* development, when the endoderm is patterned, Wnt8 and Bmp function in autoregulatory and feed-forward signaling loops which reinforce their own ligand expression as well as that of each other (Hoppler and Moon, 1998; Nakamura et al., 2016; Stevens et al., 2017). It is likely that the reduction in Wnt antagonist expression caused by Rfx6 depletion allows these Wnt8/Bmp regulatory loops to become hyperactivated, expanding the *wnt8* and *bmp* ligand expression domains into the posterior foregut. It is possible that other posterior foregut TFs downstream of Rfx6 also help restrict Wnt/Bmp ligand expression, and Rfx6 may also modulate Wnt and BMP activity at the level of enhancers. Published ChIP-seq analysis of *Xenopus* foregut tissue (Stevens et al., 2017) demonstrated that β-catenin and Smad1 bind to the same Rxf6-regulated *cdx2* and *nkx2-6* enhancers. Thus, signaling effectors and lineage-promoting TFs may converge on the same cis-regulatory elements to refine gene expression boundaries.

### Rxf6 functions as both a transcriptional activator and repressor

We demonstrated that Rxf6 can directly activate and repress key patterning genes. The RFX family of TFs can form homodimers or heterodimers amongst themselves, physically associate with co-activator or co-repressor proteins [including histone deacetylases (HDACs)] (Aftab et al., 2008; Sugiaman-Trapman et al., 2018; Xu et al., 2006; Seguin-Estevez et al., 2009; Watts et al., 2011; Didon et al., 2013; Quigley and Kintner, 2017; Lemeille et al., 2020). It is likely that interactions with RFX family members, cofactors and other TFs influence whether RFX6 functions as an activator or a repressor in a target-specific manner. Interestingly, *RFX6* variants observed in Mitchell–Riley syndrome are present not only in the DNA-binding domain, but also in the dimerization domain and largely uncharacterized N- and C-terminal domains (Concepcion et al., 2014; Chandra et al., 2014; Skopkova et al., 2016; Patel et al., 2017; Akiba et al., 2020; Imaki et al., 2021; Sanchez et al., 2024), suggesting that phenotypes may involve disruptions in protein-protein cofactor interactions. Indeed, the human Mitchell–Riley Syndrome iPSC study (Trott et al., 2020) re-analyzed in this work carries a mutation that truncates RFX6 before the dimerization domain but leaves the DNA-binding domain intact (Fig. S9A). Our work here should provide a helpful starting point for future studies examining multi-protein enhancer complexes, target-specific regulation and genotype-phenotype relationships.

### Implications for Mitchell–Riley syndrome

In humans, mutations in *RFX6* result in Mitchell–Riley Syndrome, a rare congenital recessive disorder characterized by variably penetrant neonatal diabetes, gall bladder agenesis, extrahepatic biliary atresia, duodenal atresia and intestinal malrotation (Mitchell et al., 2004). While previous studies in zebrafish (Soyer et al., 2010), *Xenopus* (Pearl et al., 2011), mouse (Smith et al., 2010) and hPSCs (Trott et al., 2020) have modeled some aspects of Mitchell–Riley Syndrome, most studies have focused on Rfx6 function in pancreas and endocrine cell development and did not address the other digestive systems phenotypes. Our results provide a mechanistic framework for understanding the diverse developmental defects of the gastrointestinal system in Mitchell–Riley Syndrome and suggest that they involve disrupted Rxf6-Wnt-Bmp patterning. Comparison of recent human embryo single-cell RNA-seq of gut tube endoderm (Li et al., 2021) to our RA/Rfx6-regulated genes suggests similar patterning occurs as early as weeks 3-4 of human gestation (Fig. S10; Table S7), and is supported by observations that multiple key endoderm TFs (*PDX1*, *ONECUT1*, *ONECUT2*, *HHEX*, *FOXE1*, *NKX2-5*, *NKX2-6*) have dysregulated levels in Mitchell–Riley Syndrome iPSC differentiation studies (Trott et al., 2020). Our recent work using patent-derived Mitchell–Riley Syndrome iPSCs differentiated into human small intestinal organoids supports this idea of abnormal early fetal patterning (Sanchez et al., 2024) but did not examine epithelial-mesenchymal interactions. In the future, it will be interesting to model Mitchell–Riley syndrome in complex foregut organoids that contain both endoderm and mesoderm, and to assess the genotype-phenotype impact of different mutations.

### Limitations of the study

Limitations of this study include the lack of available ChIP-grade anti-RFX6 antibodies to characterize genomic binding directly and identify cofactors that physically interact with Rfx6, which could differ in locus-specific contexts. In addition, the hPSC foregut cultures generated in this study only generate endoderm tissue and therefore lack the ability to study mesoderm-endoderm paracrine signaling. While the analysis of human and frog RA-regulated genes in Fig. 1 might shed some insight into ‘species-specific’ RA responses, the symbol matching used in this analysis probably underestimates the ‘functional’ conservation in RA responses. Different members of the same gene family were frequently regulated in the same way: for example, *hoxc3* and *hoxd4* were RA-induced in *Xenopus*, whereas *HOXB3* and *HOXC4* were upregulated by RA in the hPSC foregut. Moreover, we do not know whether the hPSC-derived foregut and the endogenous *Xenopus* foregut are exactly the same developmental stage, and the *in vivo* tissue likely experiences additional signals that the *in vitro* human tissue does not.

## MATERIALS AND METHODS

### *Xenopus* methods

*Xenopus* experiments were performed according to Cincinnati Children's Hospital Institutional Animal Care and Use Committee (IACUC) protocol IACUC2025-0040. Adult WT *X. laevis* (NXR_0031, *Xla.NXR-WT^NXR^*) adult transgenic *X. laevis* Wnt reporter [NXR_0064, *Xla.Tg(WntREs:dEGFP)^Vlemx^*] and adult WT *X. tropicalis* (NXR_1018, *Xtr.NigerianSt549*) frogs were purchased from the National *Xenopus* Resource Center (NXR, Woods Hole, MA, USA; RRID:SCR_013731). Ovulation, *in-vitro* fertilization, and natural mating of *X. laevis* and *X. tropicalis* were performed as previously described (Sive and Harland, 2023). Embryos were staged by the NF normal table of development (Nieuwkoop and Faber, 1994).

Previously validated translation blocking antisense morpholino oligos (MO) against *Xenopus* Rfx6 (Pearl et al., 2011; Sanchez et al., 2024) or Aldh1a2 (Strate et al., 2009; Rankin et al., 2021) were purchased from GeneTools. MO sequences and injection amounts were as follows: *X. laevis* Rfx6-MO: 5′-AATTGGCATTTCACCGGGTTCAGGC-3′; a negative control Rfx6 mismatch (MM MO) with five bases altered: 5′-AATaGGgATTTgACCcGGTTCAcGC-3′ (mismatch bases indicated in lowercase and underlined); 2 ng of Rfx6 MO or control mismatch MO injected per *X. laevis* blastomere at the two-cell stage; *X. tropicalis* Aldh1a2 MO: 5′-GCATCTCGATTTTACTGGAAGTCAT-3′; 0.3 ng injected per *X. tropicalis* blastomere at the two-cell stage; *X. laevis* Aldh1a2 MO: 5′-GCATCTCTATTTTACTGGAAGTCAT-3′; 2 ng per *X. laevis* blastomere injected at the two-cell stage.

#### *Xenopus* explant dissections for bulk RNA-seq

Foregut explants were dissected from sibling embryos in 1×MBS (Sive et al., 2000)+50 μg/ml gentamycin sulfate (gent; MP Biochemicals, 1676045). When removing the mesoderm/ectoderm, treatment with 10 units/ml dispase (Corning Life Sciences, 354235) for 5-10 min at room temperature was used as needed to aid germ layer separation. Explants were cultured in 0.5× MBS +0.2% fatty-acid-free BSA (Fisher, BP9704)+50 µg/ml gent with the following concentrations of factors: 25 nM RA (Sigma-Aldrich, R2625) for endoderm only explants; 1 µM BMS493 (Tocris, 3509) for mesoderm+endoderm explants; both were dissolved in DMSO (Thermo Fisher Scientific, BP231) and an equivalent amount of DMSO was used as vehicle control. Explants were staged according to the development of sibling whole embryos cultured at the same temperature (16°C). Three biological replicates (*N*=3) each containing *n*=8 explants, were frozen on dry ice in 200 µl Trizol (Thermo Fisher Scientific, 15596018) and stored at −80°C before RNA extraction using the Direct-zol RNA miniprep plus kit (ZymoResearch, R2070). Library preparation and sequencing was performed by the Cincinnati Children's Hospital Medical Center (CCHMC) Genomics Sequencing Facility. Briefly,150-300 ng of total RNA determined by Invitrogen QubitTM high-sensitivity spectrofluorometric measurement was poly-A selected and reverse transcribed using Illumina's TruSeq stranded mRNA library preparation kit. Each sample was fitted with one of 96 adapters containing a different eight base molecular barcode for high level multiplexing. After 15 cycles of PCR amplification, completed libraries were sequenced on an Illumina NovaSeqTM 6000, generating 20 million or more high quality 100 base long paired end reads per sample.

#### Bulk RNA-seq analysis

Raw reads were quality checked using FastQC and adapters/low-quality reads were removed using Trimmomatic (Bolger et al., 2014) and reads were mapped to the *X. laevis* v10 or *X. tropicalis* v 9.0 genomes, obtained from www.Xenbase.org (Fisher et al., 2023). The TMP (transcripts per million) and count quantification were performed using RSEM with bowtie2. Differentially expressed genes were identified with the edgeR package (Robinson et al., 2010) using an adjusted *P*-value <0.05, FDR 0.05 and a Log2 fold-change ≥0.5 (Fig. 3) or ≥0.5 (Fig. 1). The enrichment analysis was performed with GSEA (Subramanian et al., 2005), with gene sets described in the text. GO analysis was performed using Enrichr (Kuleshov et al., 2016).

#### RFX6 direct target assays

Generation of the DEX inducible (Kolm and Sive, 1995; Sinner et al., 2004) human RFX6 construct (pT7Ts-GR-RFX6) has been previously described (Sanchez et al., 2024). To make messenger RNA for injection, the XbaI-linearized plasmid template was used in Ambion mMessage mMachine T7 mRNA synthesis reactions (Thermo Fisher Scientific, AM1344). Then 100 pg GR-*RFX6* mRNA was injected at the 8/16-cell stages into a ventral-vegetal blastomere to target the hindgut or dorsal-vegetal blastomere to target foregut. Explants were dissected in 1× MBS+50 μg/ml gent and cultured in 0.5× MBS+0.2% fatty acid-free bovine serum albumin (Fisher Scientific, BP9704)+50 μg/ml gent with the following concentrations of factors: 1 μM DEX (Cell Signaling Technology, 14776); 1 μM CHX (Sigma-Aldrich, C4859) both dissolved in DMSO. The equivalent amount of DMSO was used as the vehicle control. In CHX experiments, explants were treated for 2 h with CHX before DEX+CHX treatment or before 25 nM RA (Sigma-Aldrich, R2625)+CHX treatments for 6 h.

For RT-qPCR on explants, three biological replicates of *n*=4 explants each were collected. RNA was extracted using the Macherey-Nagel Nucleospin Isolation Kit according to the manufacturer's instructions and 500 ng RNA was used in cDNA synthesis reactions using Superscript Vilo Mastermix (Thermo Fisher Scientific, 11755050). qPCR reactions were carried out using PowerUp Mastermix (Thermo Fisher Scientific, A25742) on an ABI QuantStudio3 machine. *Xenopus* RT-qPCR primer sequences are listed in Table S8. Relative expression, normalized to the housekeeping gene *odc1* and then set relative to uninjected, untreated explants, was determined using the 2^−ΔΔCt^ method. Graphs display the average 2^−ΔΔCt^ value±standard deviation. Statistical significance (*P*<0.05) was determined using parametric two-tailed paired *t*-tests, relative to uninjected, untreated explants. Each black dot in the RT-qPCR graphs represents an independent biological replicate.

#### *In situ* hybridization

*In situ* hybridization of *Xenopus* embryos was performed as previously described (Sive et al., 2000) with minor modifications. Briefly, embryos were fixed overnight (12-20 h) at 4°C in MEMFA (0.1 M MOPS, 2 mM EGTA, 1 mM MgSO_4_, 3.7% formaldehyde). Embryos were bisected (as needed) in MEM salts only (MEMFA without the formaldehyde), refixed in MEMFA for 20 min, and then dehydrated directly into 100% ethanol, washed four to six times in 100% ethanol and stored at −20°C in 100% ethanol for at least 24 h prior to *in situ* hybridization. Proteinase K (Thermo Fisher Scientific, AM2548) on day 1 was used at 2.5 μg/ml for 10 min on stage NF15 and NF25 embryos and 5 μg/ml for 10 min on NF33 embryos; hybridization buffer included 0.1% SDS; the RNase step was omitted; and anti-digoxygenin (DIG)-alkaline phosphatase antibody (Sigma-Aldrich, 11093274910) used at 1:5000 in MAB blocking buffer [100 mM maleic acid, 150 mM NaCl, pH 7.5, 20% heat-inactivated lamb serum (Gibco, 16070096), 2% blocking reagent (Sigma-Aldrich, 11096176001)]. Full-length anti-sense DIG-labeled *in situ* probes were generated using linearized plasmid cDNA templates with 10× DIG RNA labeling mix (Sigma-Aldrich, 11277073910) according to the manufacturer's instructions.

#### Immunofluorescence

Embryos were fixed in MEMFA containing 2.7% methanol-free formaldehyde (Thermo Fisher Scientific, 28906) for 2 h at 4°C, dehydrated directly into ice-cold Dent's post-fixative (80% methanol/20% DMSO), washed five times in Dent's, and stored in Dent's at −20°C for at least 24 h. Embryos were serially rehydrated for 10 min per wash at room temperature in a graded methanol series (75%, 40%, 25%), and finally into PBS +0.1% TritonX-100 (PBSTr) and were either bisected sagittally through the midline (NF25) or in a transverse plane through the pharynx and midgut (NF33) using a fine razor (EMS 72002-01) on a 2% agarose-coated dish in PBSTr to create samples used for staining. Sample were blocked for 1-2 h in PBSTr +10% normal donkey serum (Jackson ImmunoResearch, 017000121)+1% DMSO at room temperature and incubated overnight at 4°C in this blocking solution+primary antibodies. The following primary antibodies were used: rabbit anti-phosopho-Smad1/5/9 (Cell Signaling Technology, 13820, 1:300); chicken anti-GFP (Aves Labs, GFP-1020, 1:1000); goat anti FOXA2 (Santa Cruz Biotechnology, sc6554; 1:500). After five 10 min washes in PBSTr, samples were incubated overnight at 4°C in PBSTr +1% DMSO+secondary antibodies, all used at 1:1000 dilution: donkey anti-chicken Alexa 488, donkey anti-rabbit Cy3, donkey anti-mouse Cy5 or donkey anti-goat Cy5 (Jackson ImmunoResearch, 703-546-155, 711-166-152, 715-175-151, 705-175-147, respectively). Samples were again washed in PBSTr, dehydrated into 100% methanol, washed five times in 100% methanol, cleared and imaged in Murray's Clear (two parts benzyl benzoate, one part benzyl alcohol) in an Ibidi two-well glass bottom chamber (Ibidi, 80287) using a Nikon A1R confocal microscope to obtain optical sections.

#### Luciferase assays

Enhancer sequences (genomic coordinates listed below) were commercially synthesized by GenScript and cloned into the pGL4.23 firefly luc2/miniP vector (Promega, E8411). We injected 150 pg of WT or mutant versions of the enhancer:firefly luciferase reporters +15 pg pRL-TK *Renilla* luciferase plasmid (Promega, E2241) into embryos at the 16/32-cell stage into the D1 vegetal region to target the foregut or into the D4 vegetal region to target the hindgut. Each biological replicate consisted of a pool of five embryos, obtained from two or three separate fertilization/injection experiments, which were frozen on dry ice in a minimal volume of 0.1× MBS and stored at −80°C. Samples were lysed in 100 μl of 100 mM TRIS-Cl (pH 7.5) by pipetting, centrifuged for 10 min at ∼13,000 ***g*** and then 50 µl of the clear supernatant lysate was used separately in firefly (Biotium, 30085-1) and *Renilla* (Biotium, 30082-1) luciferase assays containing 200 µl substrate, 10 s measurement time, using a Promega Glomax 96-well plate reader luminometer. Relative luciferase activity was determined by normalizing firefly to *Renilla* levels for each sample. Graphs show the average relative luciferase activity±standard deviation, with black dots showing values of biological replicates. Statistical significance was determined by parametric two-tailed paired *t*-test (*P≤0.05). The following enhancer genomic sequence co-ordinate sequences were synthesized: human *RFX6* intron14: hg19_chr6:117245090-117245788 (699 bp); human *SFRP5* upstream enhancer: hg19_chr10:99548699-99549100 (401 bp); human *CDX2* intron 1 enhancer: hg19_chr13:28540120-28541224 (1105 bp); human *NKX2-6* downstream enhancer: hg19_chr8:23556228-23556699 (472 bp).

#### Transgenesis

Transgenesis was carried out using the I-SceI meganuclease technique, which has a known efficiency ranging from ∼2%-20% (Ogino et al., 2006; Pan et al., 2006; Rankin et al., 2021). Enhancer fragments were commercially synthesized (Genscript) upstream of a minimal TATA box promoter (Tran et al., 2010) flanked by the 250 bp chick β-globin HS4 insulator (Rankin et al., 2021) and cloned into the pI-SceI-d2EGFP plasmid (Addgene plasmid 32674). The meganuclease reaction contained 400 ng DNA, 5 µl I-SceI enzyme (New England Biolabs, R0694S) in an 80 µl total reaction volume and was incubated at 37°C for at least 45 min prior to injection. We injected 10 nl of this reaction into one- to two-cell embryos. Transgenic efficiencies were as follows: human *RFX6* intron14:GFP 11/91 (∼12%); human *SFRP5:GFP* 9/62 (∼14%); human *CDX2 intron1:GFP* 14/49 (∼28%); human *NKX2-6:GFP* 13/60 (∼21%). As a negative control, 0/163 embryos were GFP-positive when injected using reactions that omitted the I-SceI enzyme.

### Human pluripotent stem cell methods

#### hPSC differentiation

Human embryonic stem cell (ESC) line WA01 (H1; WiCell) was maintained in feeder-free conditions on Matrigel (BD Biosciences) in mTesR1 media (StemCell Technologies). DE differentiation was performed as previously described (Rankin et al., 2016). Briefly, ESCs were dissociated into single cell suspension using accutase and plated into a 24-well plate in mTesR1 with 10 µM ROCK inhibitor Y-27632. On the following day, cells were exposed to Activin A (100 ng/ml; Cell Guidance Systems) for 3 days in RPMI 1640 media (Invitrogen, 61870036) that contained increasing concentrations of 0%, 0.2% and 2.0% defined fetal bovine serum (Invitrogen, A5669501) on each day. BMP4 (10 ng/ml; R&D Systems) was added on the first day of DE induction. DE was patterned into foregut endoderm by 72 h culture in RPMI 1640 media with 2.0% dFBS+Noggin (200 ng/ml; R&D Systems). All-trans RA (2 µM; Sigma-Aldrich, R2625) was added to the media for 24 h on the final day of Noggin treatment (day 5-6). RNA from separate wells was harvested for bulk RNA-seq using the Macherey-Nagel Nucleospin RNA isolation kit (740955.250) according to the manufacturer's instructions. Library preparation and sequencing was performed by the CCHMC Genomics Sequencing Facility as described in the *Xenopus* RNA-seq section above.

#### ATAC-seq of hPSCs

ATAC-seq was performed as previously described (Buenrostro et al., 2015; Mukherjee et al., 2022). Briefly, 50,000 cells were collected following differentiation to the desired stage and lysed in 50 ml of ATAC-lysis buffer (10 mM Tris-HCl, pH 7.4. 10 mM NaCl, 3 mM MgCl_2_, 0.1% NP-40) to obtain a nuclei preparation. Centrifugation steps were performed at 4°C at 2000 rpm (400 ***g***). The nuclei pellet was then resuspended in the 50 µl of transposition reaction mix (25 µl Tagment DNA buffer, 2.5 µl TD Tn5 Transposase enzyme, 22.5 µl nuclease-free water) (Illumina, 20034197) and incubated at 37°C for 30 min on a thermomixer with constant gentle shaking at 1000 rpm. Immediately after transposition, DNA was purified using a Qiagen MinElute PCR Purification kit (Qiagen, 28004) and eluted in 10 ml Elution Buffer. Library preparation was performed by the CCHMC Genomics Sequencing Facility, briefly as follows: transposed DNA was amplified in a reaction with 25 µl NEBNext High-Fidelity 2× PCR Mastermix (New England Biolabs, M0541L) and custom 25 µm Nextera PCR Primers (Ad1_noMx universal primer, 0.5 µm Ad2.x indexing primer). PCR was performed as follows: 1 cycle of 72°C for 5 min, 98°C for 30 s, 5 cycles of 98°C for 10 s, 63°C for 30 s, 72°C for 1 min. Then, 5 µl of the amplified DNA was used to perform qPCR to determine the optimal number of additional cycles to prevent amplification saturation of DNA libraries. In all cases, either four or five additional cycles of PCR was performed at: 98°C for 10 s, 63°C for 30 s and 72°C for 1 min. Double size selection of amplified libraries (0.5× – left sided, 1.8× – right sided) was performed using AMPure XP beads and DNA was eluted in a final volume of 20 µl in 0.1× TE buffer. Purified libraries were sequenced on an Illumina NextSeq 500 instrument by the CCHMC Genomics Sequencing Facility.

#### Human RNA-seq analysis methods

Raw reads were quality checked using FastQC and adapters/low-quality reads were removed with Trimmomatic (Bolger et al., 2014). Gene count quantification and read mapping to the *Homo sapiens* hg38 genome (GRCh38) were performed with STAR 2.7.9 aligner (Dobin et al., 2013). Differentially expressed genes were identified with the edgeR package (Robinson et al., 2010) using an adjusted *P*-value <0.05 and a Log2 fold-change ≥1.5.

#### Previously published datasets used for analysis

GEO accession numbers for previously published ChIP-seq and ATAC-seq data sets utilized in this study are as follows. *Xenopus* ChIP-seq: GSE87652 (Stevens et al., 2017). Human ChIP-seq: GSE114102 (Lee et al., 2019); GSE104840 (Vinckier et al., 2020); GSE148368 (Geusz et al., 2021); GSE182842 (Mukherjee et al., 2022). Human ATAC-seq: GSE222443 (Kearns et al., 2023); GSE206586 (Cipriano et al., 2024); GSE114101 (Lee et al., 2019). Bigwig files for these datasets were downloaded from Xenbase (http://www.xenbase.org/; RRID:SCR_003280) or GEO and viewed using the Integrated Genomics Viewer browser, version IgV_2.5.2 (Broad Institute; https://software.broadinstitute.org/software/igv/).

## Supplementary Material



10.1242/develop.205552_sup1 Supplementary information

Table S1. DEGs related to Fig. 1

Table S2. Motif analysis of Rfx6 intron 14 enhancer

Table S3. DEGs related to Fig3. RA Rfx6 LOF NF25 NF33

Table S4. DEGs related to Fig3 GSEA

Table S5. DEG list related to Fig S5

Table S6. DEGs from Trott et al MRS iPSC

Table S7. Gene lists Human Embryo scRNA_seq

Table S8. *Xenopus RTqPCR primers*
