# Supplementary material for: A retinoic acid-Rfx6 cascade modulates Wnt/Bmp-dependent gut tube patterning: insight into the molecular basis of Mitchell–Riley Syndrome
Source: Development. 2026 Sep 29;153(18):dev205552. doi: 10.1242/dev.205552 (PMC13677009; doi:10.1242/dev.205552)
Supplement: Supplementary file 1 [file develop-153-205552-s1.pdf]

Supplemental Fig.S1 related to Figure 1.

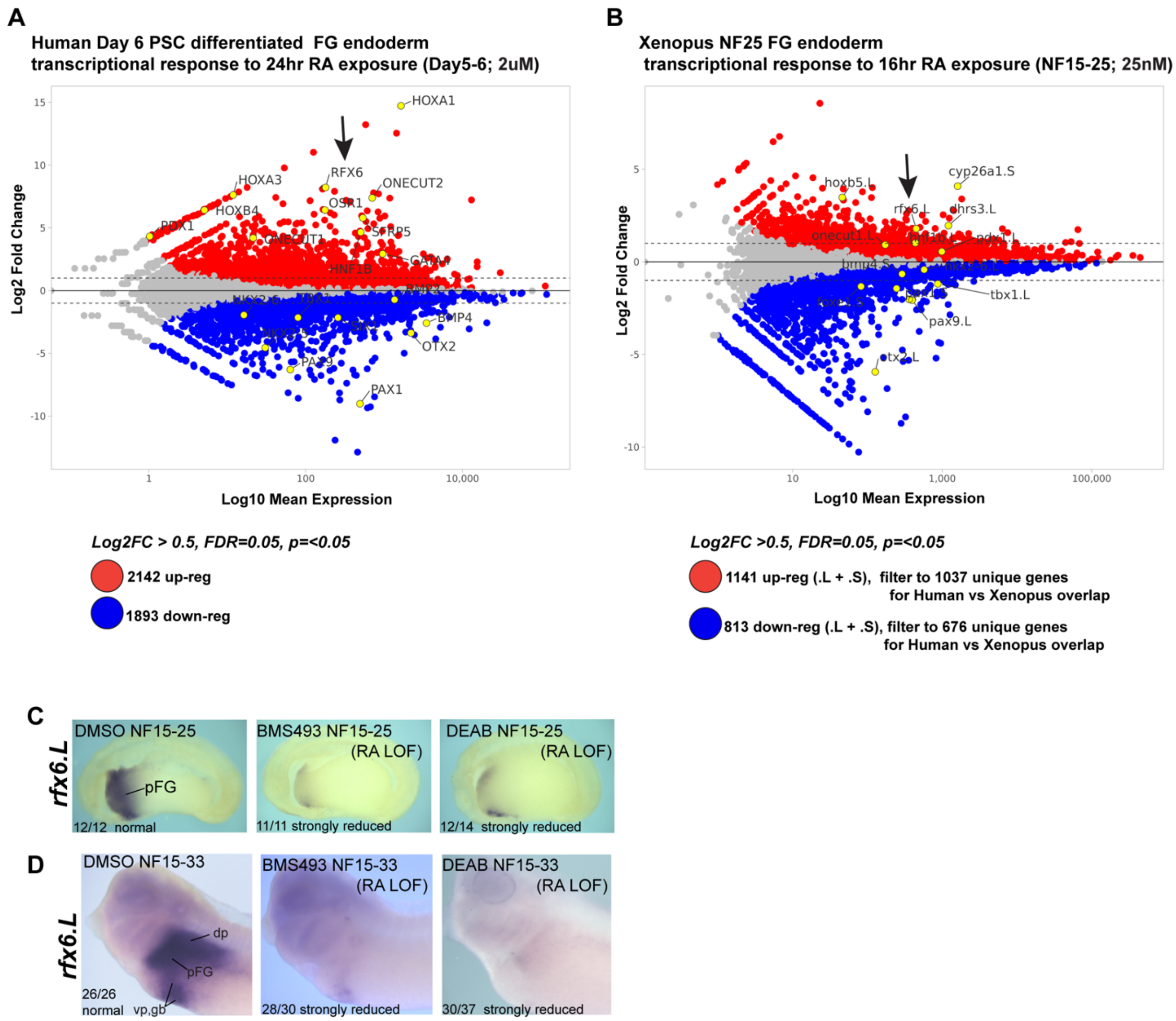

Fig. S1. *RFX6* is a conserved RA-activated gene, and endogenous RA signaling is required for *rfx6* expression in *X. laevis*.

(A-B) MA plots of differentially expressed genes after RA exposure in human and *Xenopus laevis* foregut endoderm. Selected genes are colored yellow for ease of visualization. Black arrow points to *Rfx6* in human and *Xenopus*. **(C,D)** In situ hybridization of *rfx6.L* expression in *X.laevis* embryos at NF25 **(C)** or NF33 **(D)** after treatment with the pan RAR antagonist BMS493 or Aldh inhibitor DEAB for the indicated times. Numbers in the lower left of the images indicate the number of embryos assayed that displayed the representative staining pattern shown.

Supplemental Figure S2 related to Figure 2.

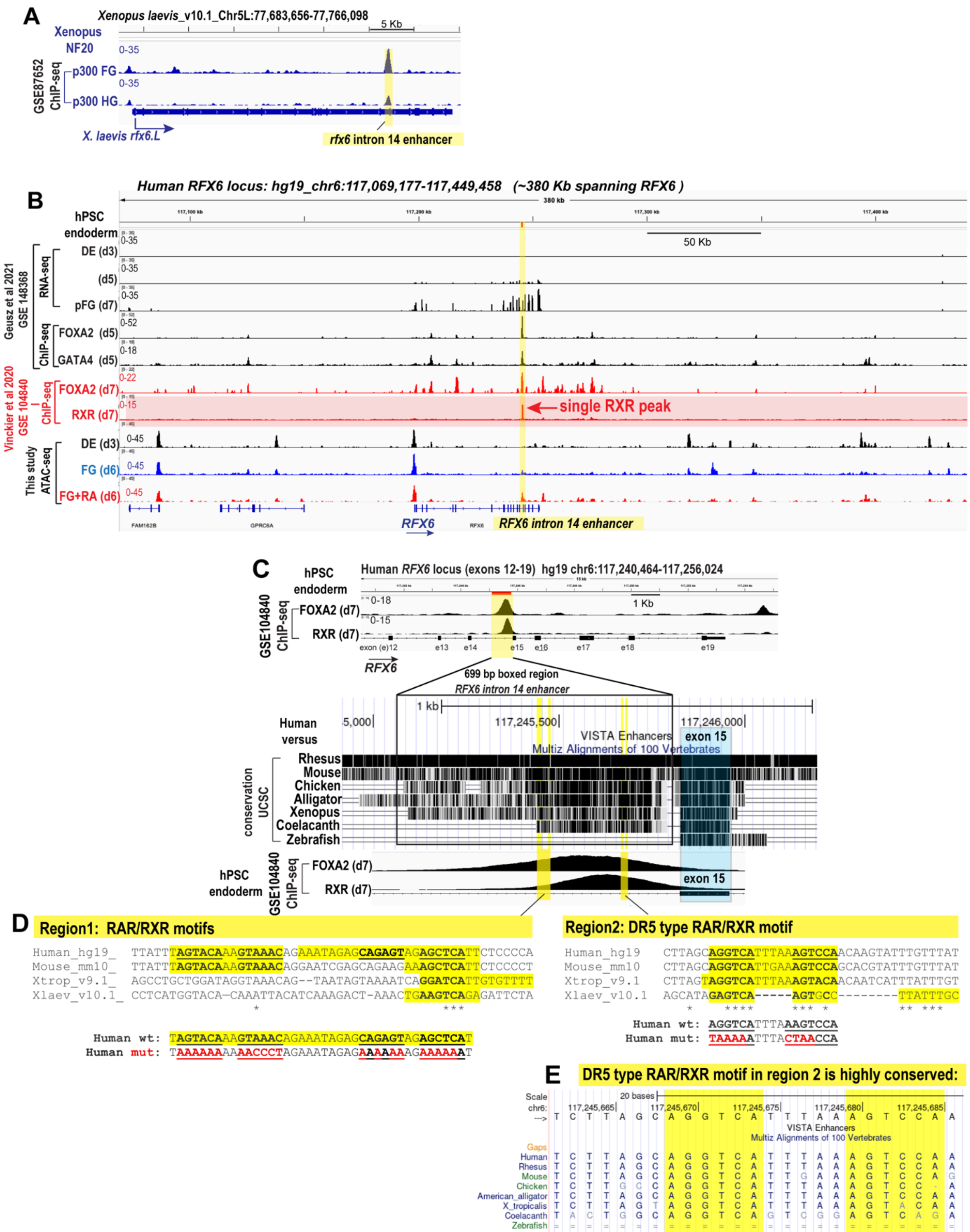

Development • Supplementary information

Fig. S2. Analysis of the conserved *RFX6* intron 14 enhancer.

(A). IgV browser view of published p300 ChIP-seq tracks of *Xenopus laevis* NF20 foregut (FG) and hindgut (HG) dissected tissue (GSE87652, Stevens et al., 2017) shows enriched binding of p300 at the conserved *rfx6.L* intron 14 enhancer region in FG as compared to HG. **(B).** IgV browser view of published RNA-seq and ChIP-seq spanning ~380 Kb around the human *RFX6* locus, in comparison to ATAC-seq generated in this study. Yellow shaded region indicates *RFX6* intron 14 enhancer. Published RNA-seq at day3 (d3), day5 (d5), and day 7 (d7) shows the temporal profile of *RFX6* expression, with binding of FOXA2 and GATA4 at day5 at the intron 14 enhancer (GSE14868; Geusz et al., 2021 wherein RA treatment was from day 5 to day 7. A single RXR binding peak (red arrowhead) is indicated in the day7 RXR ChIP-seq data (GSE104840, Vinckier et al., 2020 wherein RA treatment was day5 to day7). **(C).** Zoom in of the *RFX6* intron 14 region showing UCSC genome browser conservation tracks and the RXR / FOXA2 ChIP-seq tracks from Vinckier et al., 2020 (GSE104840). **(D).** CisBP motif analysis (Weirauch et al., 2014) identified the RXR/RAR motifs in the RXR-ChIP-seq region shown with yellow shading. Mutations made to the RXR/RAR motifs in the human luciferase reporter construct used in main Fig.2 are indicated in red. **(E.)** UCSC genome browser conservation tracks show the DR5 (direct repeat separated by 5bp) type RXR/RAR motif is highly conserved from Human to Coelacanth.

Development • Supplementary information

Supplemental Fig.S3 related to Figure 3: GSEA and GO analysis of RA/Rfx6 co-regulated genes

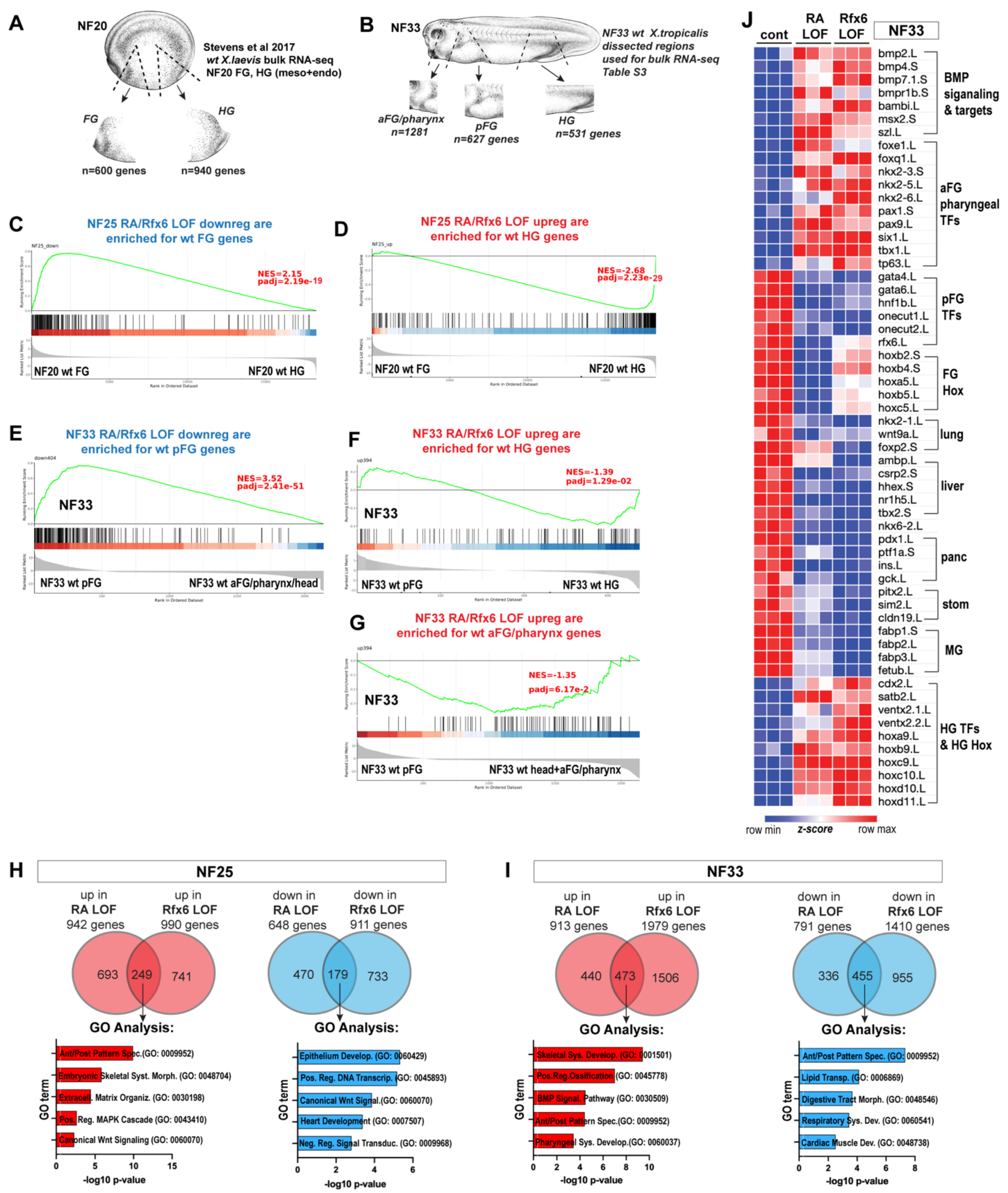

Development • Supplementary information

Fig. S3. GSEA and GO analysis of RA/Rfx6 commonly regulated genes.

(A) Schematic of dissected tissues used for GSEA analysis. **(C-G)** GSEA enrichment plots. In C and D**,** the NF25 RA/Rfx6 co-regulated genes identified in this study were compared to (A) NF20 foregut and hindgut gene sets from Stevens et al., 2017. In E,F,G, the RA/Rfx6 NF33 co-regulated genes were compared to (B) NF33 dissected regional domain gene sets generated in this study. **(H,I)** Gene ontology (GO) analyses performed using Enrichr (Kuleshov et al., 2016; of the indicated RA/Rfx6 commonly up or down-regulated genes lists at NF25 and NF33. **(J)** Heat map of NF33 timepoint demonstrates continued up-regulation of hindgut transcriptions factors (TFs) and posterior Hox genes in both RA LOF and Rfx6 LOF.

Development • Supplementary information

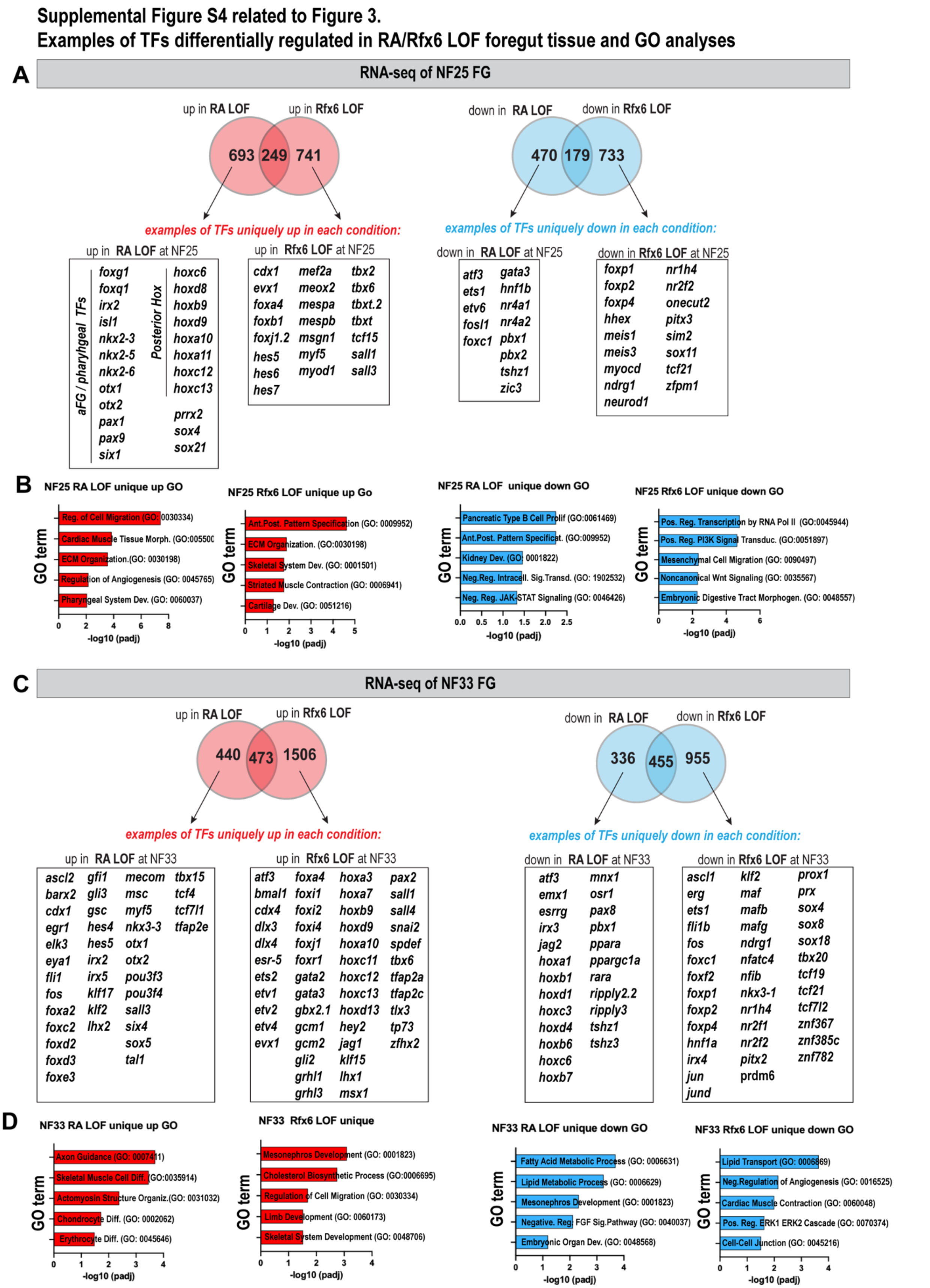

Fig. S4. Examples of TFs differentially regulated by RA/Rfx6 and gene ontology analyses. Gene lists used for the supplemental figure S4 overlaps are provided in Table S3**. (A)** Overlap analysis at NF25 and (**C**) analysis at NF33, with examples of TFs uniquely regulated in each condition. GO analyses in (**B,D**) were performed using table S5 gene lists that contain all uniquely differentially expressed genes in each condition.

Supplemental Figure S5 related to Figure 3.

A. Genes activated by RA / Rfx6 (comparative analysis of Fig.1 + Fig.3)

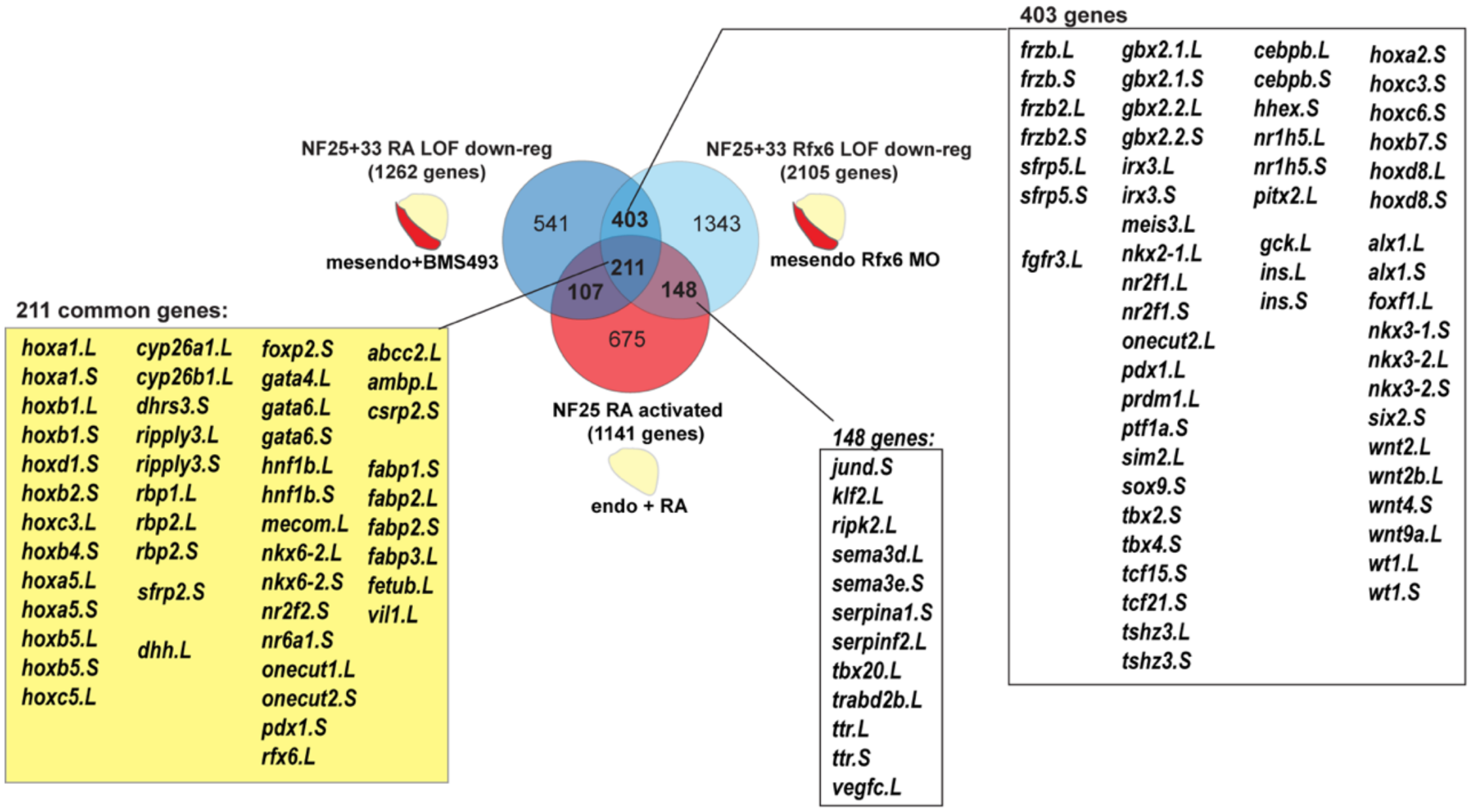

B. Genes suppressed by RA / Rfx6 (comparative analysis of Fig.1 + Fig.3)

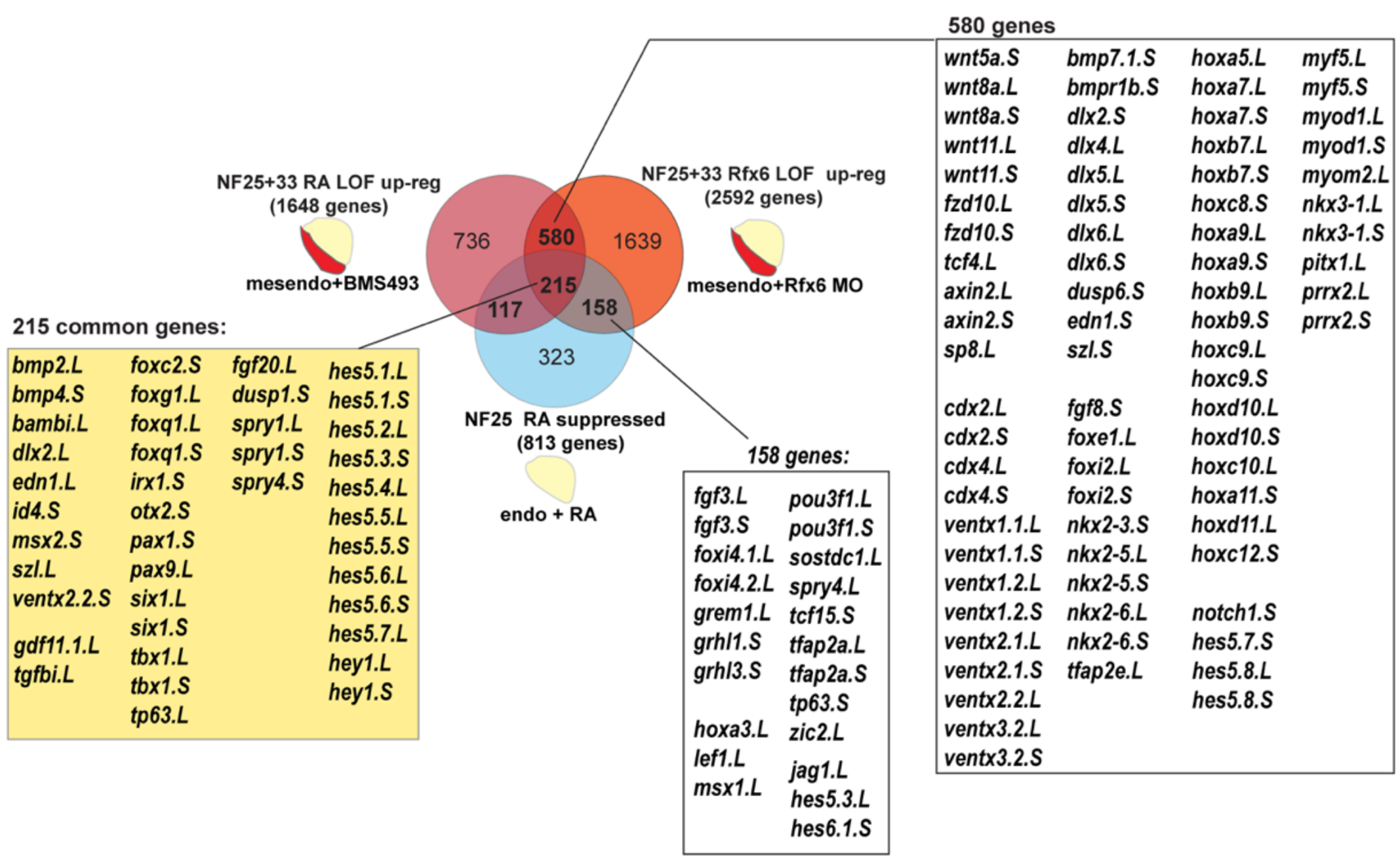

Fig. S5. Comparison of Xenopus genes identified by RA treatment (Figure 1) to Xenopus genes identified by RA and Rfx6 loss-of-function (Figure 3). Gene lists used for this figure are provided in Table S5, wherein NF25+NF33 timepoints were combined for RA LOF and Rfx6 LOF to generate the gene lists. **(A)** Genes activated by RA and/or Rfx6. **(B)** Genes suppressed by RA and/or Rfx6.

Development • Supplementary information

Supplemental Figure S6 related to Figure 4

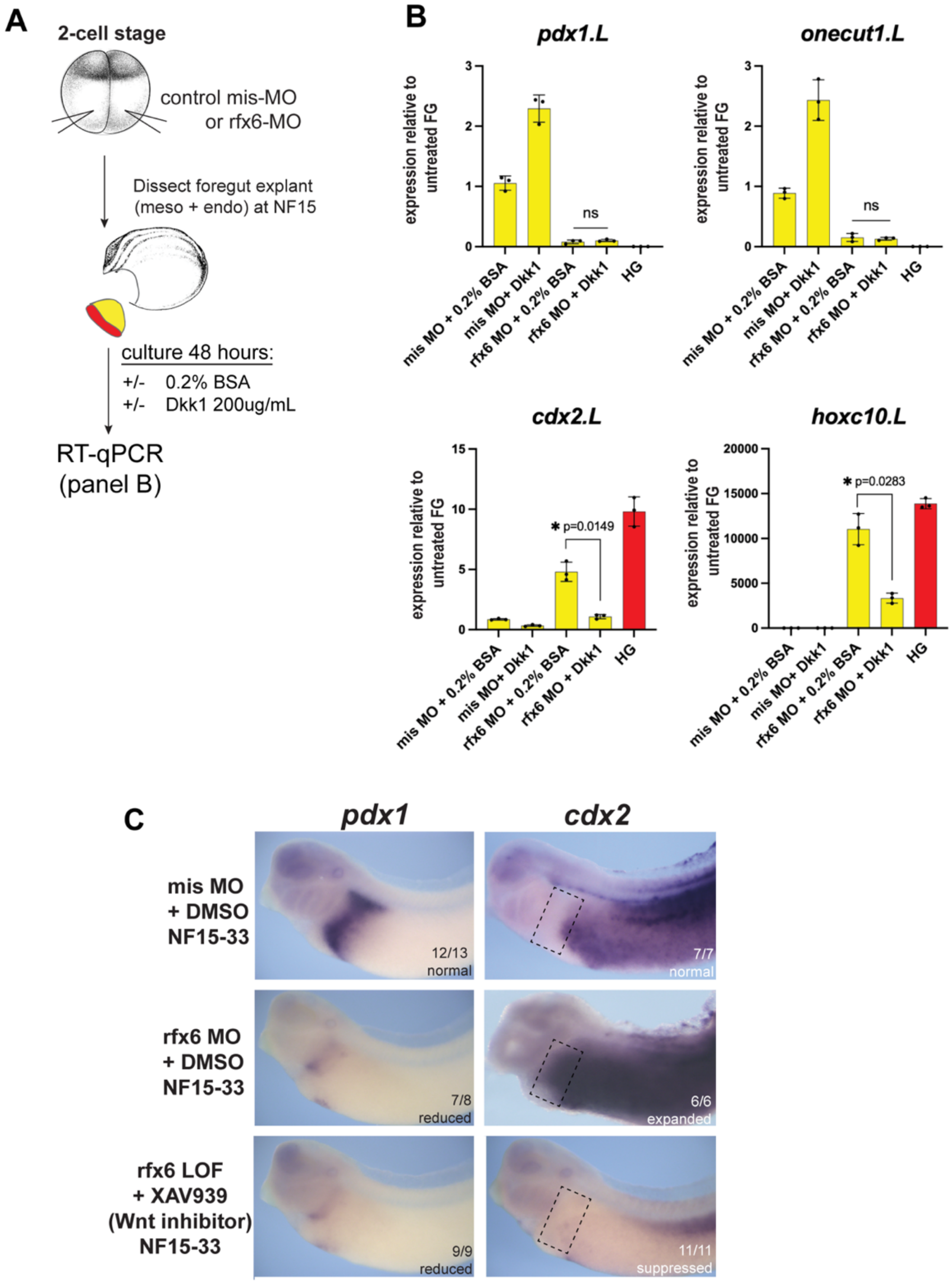

Fig. S6. Inhibiting canonical Wnt activity in Rfx6 LOF explants or embryos can suppress hindgut markers *cdx2, hoxc10* but does not rescue expression of *pdx1* or *onecut1*.

(A,B) Experimental schematic and analysis of gene expression in foregut explants by RT-qPCR. **(C)** In situ hybridization of *pdx1* and *cdx2* at NF33 in the indicated conditions. Boxed area in *cdx2* assayed embryos indicates foregut domain that *cdx2* is abnormally expanded into in Rfx6 LOF.

Supplemental Figure S7 related to Figure 5

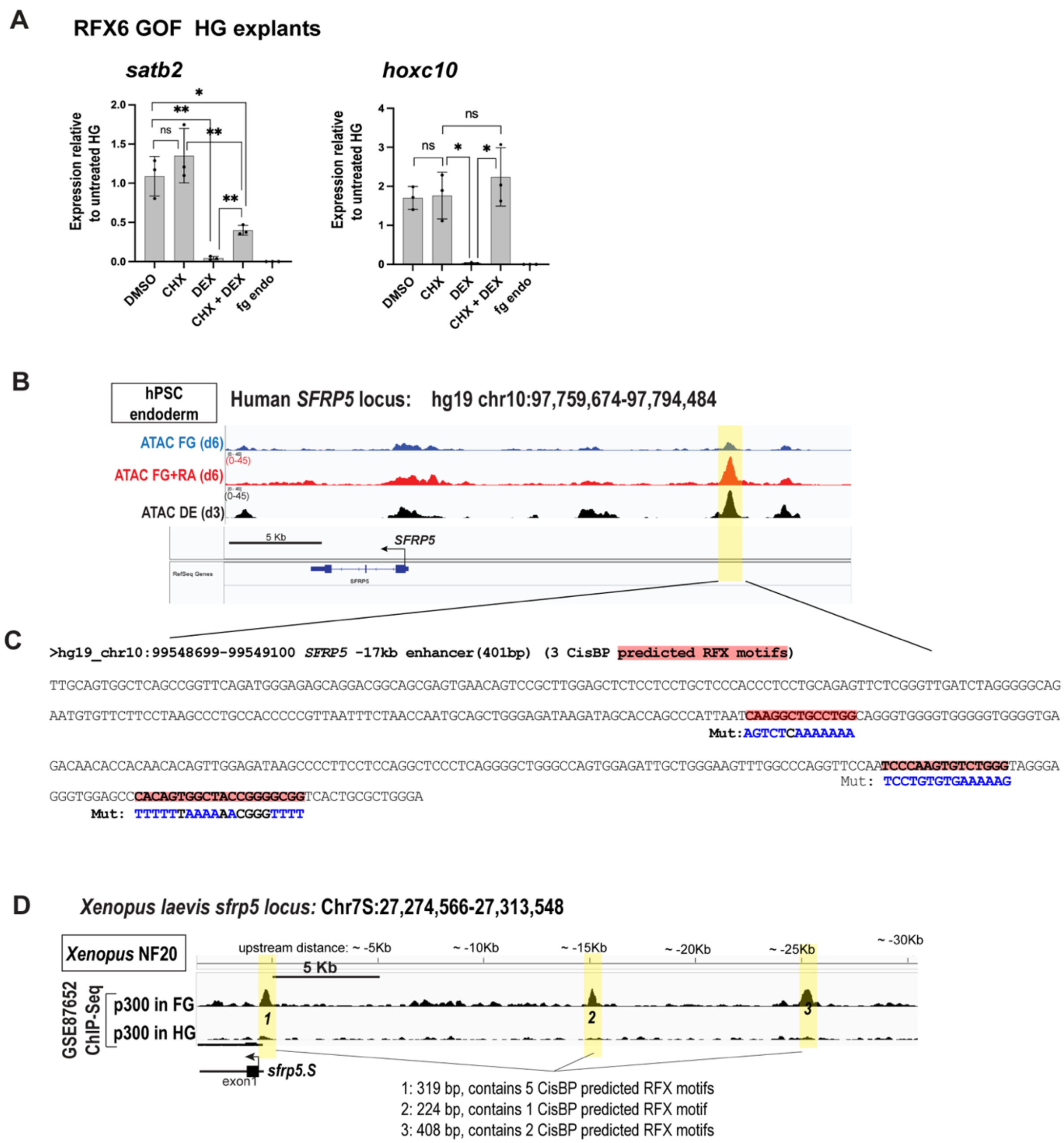

Fig. S7. related to Figure 5. Analysis of the human *SFRP5* -17Kb upstream *and X. laevis sfrp5* upstream regions.

(A) RT-qPCR analysis of *satb2 and hoxc10* expression in RFX6 gain-of-function (GOF) hindgut explants. *p=<0.05, p**=p<0.01, students 2-tailed parametric t-test. **(B)** Analysis of human *SFRP5* upstream enhancer located ~17kb upstream of TSS. The highlighted yellow region indicates the location of the enhancer, a 401bp sequence. **(C)** In the zoom-in of the enhancer, CisBP-predicted binding motifs for RFX are highlighted in red, and mutations at those sites for mutant luciferase reporter experiments in main Fig.5 are shown in blue. **(D)** Analysis of *X.laevis sfrp5* upstream region for binding of p300, a mark of active enhancers, in published ChIP-seq (GSE87652) of NF20 foregut (FG) and hindgut (HG) tissue. Highlighted in yellow are three p300 bound regions that contain the indicated RFX motifs.

Development • Supplementary information

Supplemental Figure S8 related to Figure 5

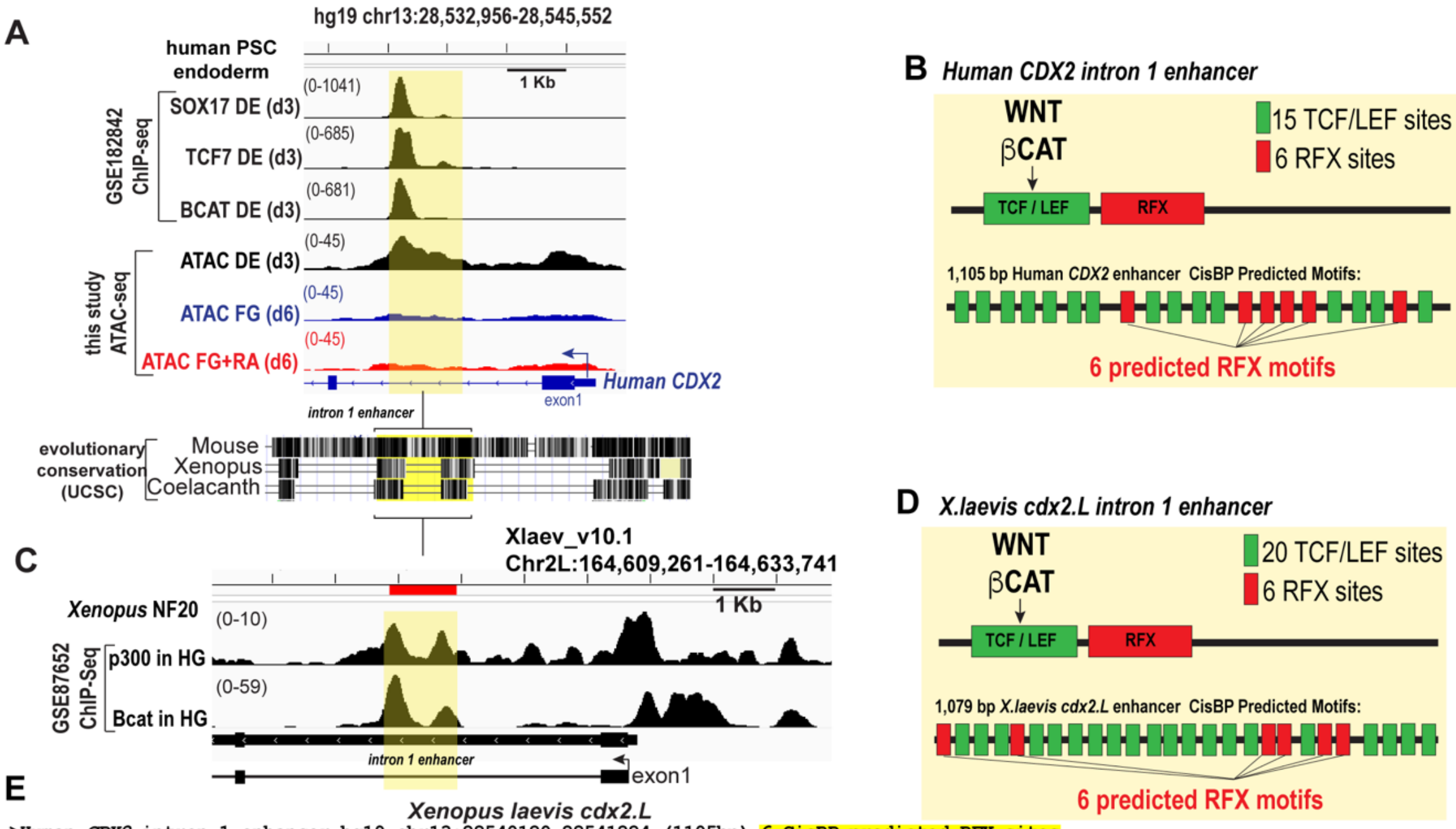

>Human *CDX2* intron 1 enhancer hg19_chr13:28540120-28541224 (1105bp) **6 CisBP predicted RFX sites**

TTGCCTGGAGGTGTTTAATGCCCCGCAGGCTGACTTTGTTCAGGTAAAACCCTTTGCAAACCACAACAAAAGCGTCCTGGGAAGATACAGGCCTGATCAGAGGGAGCTCCCCCGAGCCGCTGAAAGGCCAACACAGCCGCA
AACAATGCAGGACAAGGCGATCTTATCAAAGAAAAGCCCTTTCAATG**CCTTAATAACAACC**ACATTCTGTTTGAATTACCACTACTGAGCAAATGGCGGCCCCGGCTTTCATCCCCCTCCCCGCCAGGCGCATTAACATAA
ACACGACCCTGCAGGCGCATTTGAATGGGGCTGCGGCCCCGACCCCGCGCCTGCTATGAAAGTAAAGGGAACTAACGCGACTTTCTCCGCGGTGGACGCTCGGACACGCGTGACCGCCTCAAACCCACTCCAGGCTGCCAT
TGGTACTCGCCCCTTTTTACAGATGAGGAAATGGAGAATCAGACCGGGTCACGCAGATAGTATCAGGCGG**GGTTGGCACCGGAGCC**TGGCTGGAGCCACAGAGGACACCTCCGGAGCTGAGGCGCAGGGCAGTTCCCTCAG
CCGAACACCCCGGGAGGGAGACTCGCCTGGAAGTGTCTC**GGAGCGACCCTTGCTCG**GGTGCTGCCCCTTCAGTGTCCCGGACGTG**CCGGGCAACG**CGATGACCAGACCCTGAGCGCTCTTCGACCCATGCCCCAAGGGCCT
TGGAGGCGCCCCACACC**CCCTGGGAACCTAGGA**GCGGGGCTATGGAGGGGAGGGATGAGAGGGACTTGTCAGAATTCATTATTGACTGCGAAAGTCACCACCCTCTTTCCGGGCCCTAAAAGAGACAATGGCAGGGCTGGG
AAGGTGTATATCAAAGCAAGCCAGGCCGCGTCCCCCCTCCACCCCCACACCTTGACAGATGACGCTCCGCAGAGGAGCCCGGCTCCGGCCCGCGGGCTGCGGCCACCCCACATTAACATCACA**AGGGCACCCGGCGCCGA**
CTGCGGCCTTGCCACCTCGGCGCAGGACTTCACAGCGGCCTCTCATCTGCTGACCCCGCGGCCTGGGCTCTGGCGGCTTCCCTCCCGCCCCCTTCCCGGCCCTCACCCCGCGGCCGCT

Mutatations made in 6 predicted RFX motifs within Human CDX2 intron 1 enhancer

RFX site 1: **CCTTAATAACAACC**
Mut site 1: **AGAGCGTAGCTCAA**

RFX site 2: **GGTTGGCACCGGAGCC**
Mut site 2: **AACGACCATGACAAAA**

RFX site 3: **GGAGCGACCCTTGCTCG**
Mut site 3: **AACAAAATTTGGAAAAA**

RFX site 4: **CCGGGCAACG**
Mut site 4: **CTAAAGGGTA**

RFX site 5: **CCCTGGGAACCTAGGA**
Mut site 5: **AAATAAACGTTAAAAA**

RFX site 6: **AGGGCACCCGGCGCCGA**
Mut site 6: **AAAATGAAAATGTAAAA**

Fig. S8. Related to Fig.5. Analysis of the *CDX2* intron 1 enhancer.

(A). IgV browser view of published ChIP-seq (GSE182842, Mukherjee et al., 2022) compared to ATAC-seq generated in this study and evolutionary conservation. 1105bp Intron 1 region used in reporter assays in main Fig.5 highlighted in yellow (hg19_chr13:28540120-28541224) **(B).** CisBP motif analysis (Weirauch et al 2014) predicted RFX (red) and TCF/LEF motifs (green) in the human 1105 bp *CDX2* intron 1 enhancer. **(C).** IgV browser view of published ChIP-seq (GSE87652, Stevens et al., 2017) show binding of p300 and B-catenin in *X. laevis* NF20 hindgut tissue at the *cdx2* intron 1 enhancer. **(D).** CisBP predicted RFX (red) and TCF/LEF motifs (green) in the *X. laevis* 1079 bp *cdx2.L* intron 1 enhancer. **(E).** The 6 predicted RFX motifs in the human *CDX2* intron1 enhancer were mutated as shown for luciferase reporter assays in main Fig.5M.

Development • Supplementary information

Supplemental Figure S9 related to Figure 6

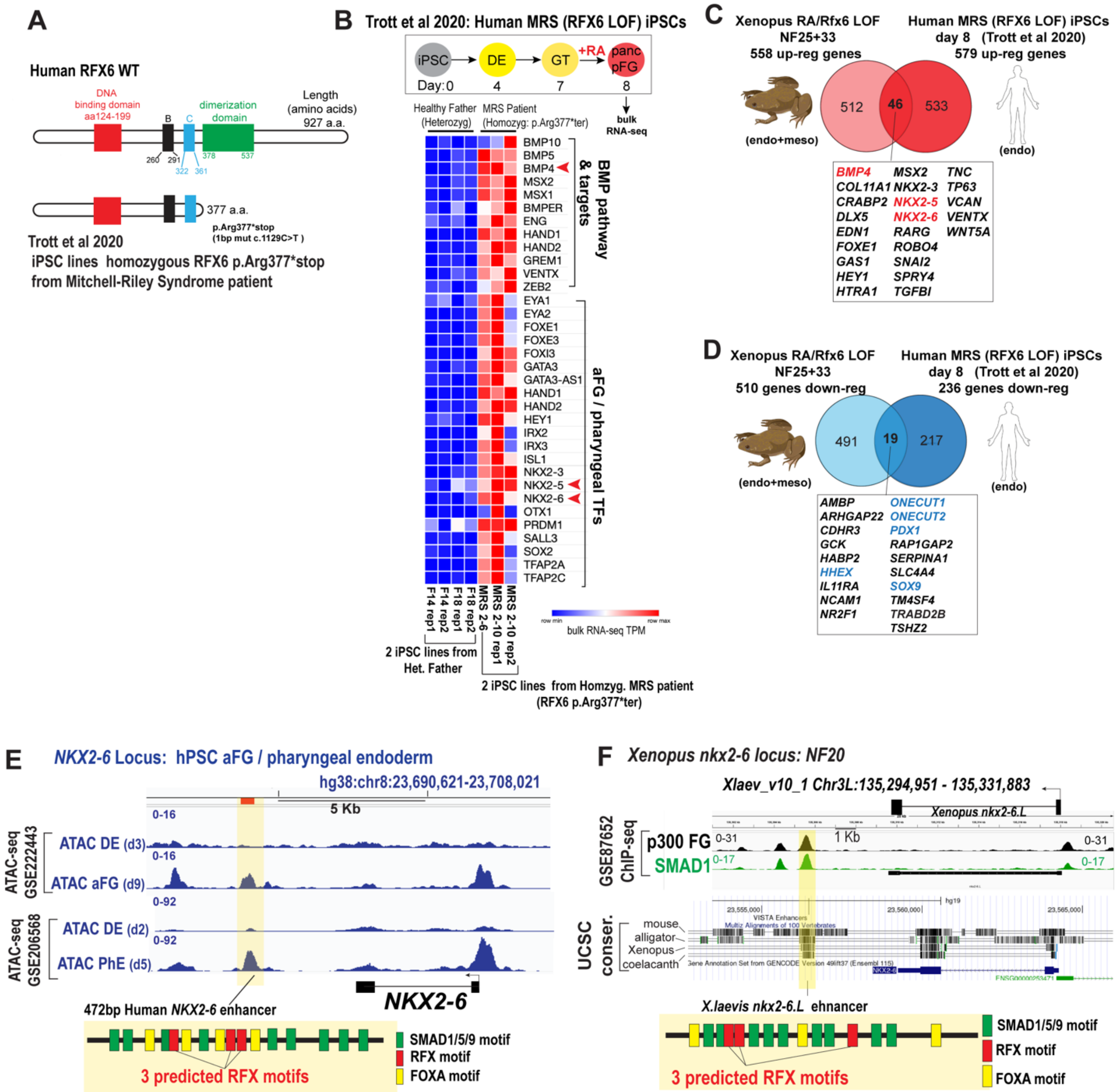

G

>hg19_dna range=chr8:23556228-23556699 *NKX2-6* enhancer (472bp) (predicted RFX6, SMAD1/5/9, FOXA)

AATTAGACTCCATCTAACTTGCAGATGGTATCAGGATGGAGCTGGCTTGGAGTGGGGCTGGGATGGGAAAGGAAAGACAGGGAGCCAGGAAGTCTGCAACTTTATTGACTTATCCACCTGGCCTAGGTGAGACCCCTAGCC

CTGGGA**CAGTCAGGAAAGGAACCT**CTGCTATCTCTCTGAGAGCTTTTTTGTTTTGTTTAGAGGTCAATTACTTCCCATTTGGGGAACTTGTGAACATTAAGAGGTGACCGGAGTGAATGATGGAGATAAGAGGCTGATTGC
Mut: **TTAAAATTCCCTTTTAAA**

TTACAGAGCG**GCTCCCTGGTAAAAGCTCT**GCAGAGGA**TAAGCAGTTGTCGGTAAGA**GACACACAAAAAGGGGCAGCTTTCAGTGCAGAGGAGAGATTACGGAGACAGAAGACAGGCAGCTGGGGGTGGGCTTGATGAGCTG
Mut:**AAATTTGAACCCTTGAAAA** Mut:**AAAAATAAAACAAATTTTT**

GGAGAGTGATGCGTTTTAAAGTGATCTTGACAGGCAGAGAGTGGGGAGA

Development • Supplementary information

Fig. S9. Related to Figure 6. Comparison of published hPSC MRS DEGs to *Xenopus* RA/Rfx6 DEGs.

(A,B). Re-analysis of Trott et al., 2020 human Mitchell-Riley Syndrome iPSC bulk RNA-seq data. The RFX6 LOF mutation in the affected patient iPSC cells is a protein truncating mutation, results in p.Arg377*stop, that is predicted to truncate RFX6 prior to the dimerization domain as shown. Heat map displays a curated list of BMP pathway genes, known BMP targets, and known anterior foregut / pharyngeal transcription factors. **(C,D).** Venn overlap analysis of Trott et al DEGs (log2FC>1.5) versus combined NF25+33 lists of RA/Rfx6 co-regulated genes from this study identifies *BMP4, NKX2-5* and *NKX2-6* as conserved targets. See table S4 for gene lists. **(E).** Re-analysis of published ATAC-seq data from two human PSC pharyngeal endoderm differentiation studies (GSE222443, Kearns et al., 2023; and GSE206568, Cipriano et al., 2024) shows the downstream *NKX2-6* enhancer region analyzed in main Fig.6 becomes accessible during hPSC differentiation (day9 or day5) as compared to the earlier definitive endoderm stage (day2 or day3). CisBP predicted RFX, SMAD1/5/9, and FOXA motifs indicated in the enhancer region cartoon. **(F).** Re-analysis of published ChIP-seq (GSE87652, Stevens et al., 2017) shows binding of p300 and Smad1 to the conserved *X.laevis nkx2-6.L* downstream enhancer. CisBP predicted RFX, SMAD1/5/9, and FOXA motifs indicated in the enhancer region cartoon. **(G)** Sequence of the human NKX2-6 downstream enhancer with CisBP predicted motifs highlighted and mutations to the 3 RFX motifs indicated in blue.

Development • Supplementary information

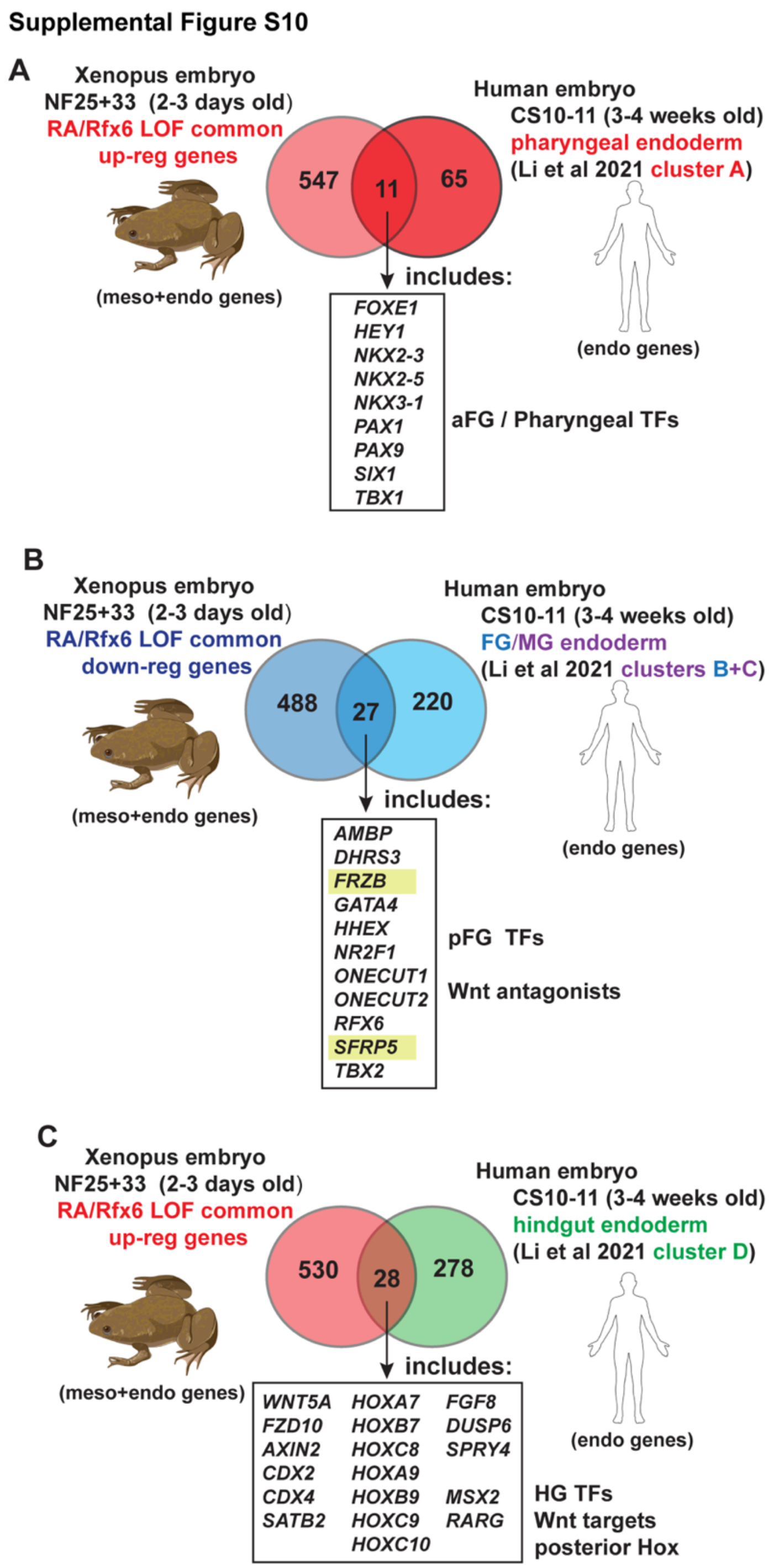

Fig. S10. Comparison of published human embryo 3-4 week old scRNA-seq gene lists to RA/Rfx6 co-regulated genes identified in this study.

(A-C) Published gene lists from human embryo 3-4 week old regional endoderm gut tube domains (Li et al., 2021; see table S5 for gene lists) were overlapped with RA/Rfx6 co-regulated genes identified in this study. Boxed genes highlight key regulators or markers of gut tube patterning from animal models.

Development • Supplementary information

Table S1. DEGs related to Fig. 1

Available for download at
https://journals.biologists.com/dev/article-lookup/doi/10.1242/dev.205552#supplementary-data

Table S2. Motif analysis of Rfx6 intron 14 enhancer

Available for download at
https://journals.biologists.com/dev/article-lookup/doi/10.1242/dev.205552#supplementary-data

Table S3. DEGs related to Fig3. RA Rfx6 LOF NF25 NF33

Available for download at
https://journals.biologists.com/dev/article-lookup/doi/10.1242/dev.205552#supplementary-data

Table S4. DEGs related to Fig3 GSEA

Available for download at
https://journals.biologists.com/dev/article-lookup/doi/10.1242/dev.205552#supplementary-data

Table S5. DEG list related to Fig S5

Available for download at
https://journals.biologists.com/dev/article-lookup/doi/10.1242/dev.205552#supplementary-data

Table S6. DEGs from Trott et al MRS iPSC

Available for download at
https://journals.biologists.com/dev/article-lookup/doi/10.1242/dev.205552#supplementary-data

Table S7. Gene lists Human Embryo scRNA_seq

Available for download at

https://journals.biologists.com/dev/article-lookup/doi/10.1242/dev.205552#supplementary-data

Table S8. ***Xenopus RTqPCR primers***

Available for download at

https://journals.biologists.com/dev/article-lookup/doi/10.1242/dev.205552#supplementary-data
