# Supplementary material for: A retinoic acid-Rfx6 cascade modulates Wnt/Bmp-dependent gut tube patterning: insight into the molecular basis of Mitchell–Riley Syndrome
Source: Development. 2026 Sep 29;153(18):dev205552. doi: 10.1242/dev.205552 (PMC13677009; doi:10.1242/dev.205552)
Supplement: Supplementary file 2 [file develop-153-205552_review_history.pdf]

A Retinoic Acid-Rfx6 cascade modulates Wnt/Bmp-dependent gut tube patterning: insight into the molecular basis of Mitchell-Riley Syndrome

Scott A. Rankin, Jacek Biesiada, Kyle E. McCracken, Riya K. Patel, Mehek Rana, David M. Luedeke, Daniel O. Kechele, James M. Wells and Aaron M. Zorn
DOI: 10.1242/dev.205552

Editor: Anna-Katerina Hadjantonakis

Review timeline
Original submission: 2 February 2026
Editorial decision: 10 March 2026
First revision received: 14 July 2026
Accepted: 4 August 2026

Original submission

First decision letter

MS ID#: dev.205552

MS TITLE: A Retinoic Acid-Rfx6 cascade modulates Wnt/Bmp-dependent gut tube patterning: insight into the molecular basis of Mitchell-Riley Syndrome

AUTHORS: Scott A. Rankin, Jacek Biesiada, Kyle E. McCracken, Riya K. Patel, Mehek Rana, David M. Luedeke, Daniel O. Kechele, James M. Wells and Aaron M. Zorn

Dear Aaron,

I have now received all the referees reports on the above manuscript, and have reached a decision. The referees' comments are appended below.

The overall evaluation is very positive and we would like to publish a revised manuscript in Development, provided that the referees' comments can be satisfactorily addressed. Please attend to all of the reviewers' comments in your revised manuscript and detail them in your point-by-point response. If you do not agree with any of their criticisms or suggestions explain clearly why this is so. If it would be helpful, you are welcome to contact us to discuss your revision in greater detail. Please send us a point-by-point response indicating your plans for addressing the referees' comments, and we will look over this and provide further guidance.

Reviewer 1

SUMMARY OF THE ADVANCE MADE IN THIS PAPER AND ITS POTENTIAL SIGNIFICANCE TO THE FIELD

In the manuscript by Rankin et al., the authors address a long-standing question in developmental biology: how different signaling pathways, such as Retinoic acid (RA), Wnt, and Bmp, and transcription factors (TF) are mechanistically integrated into regulatory networks that control organogenesis. The study focuses on the patterning of the foregut endoderm into distinct organ primordia along the antero-posterior axis. Using a multispecies approach, combining Xenopus embryos and human stem cell models, the authors identified a conserved RA-responsive

© 2026. Published by The Company of Biologists under the terms of the Creative Commons Attribution License (https://creativecommons.org/licenses/by/4.0/).

transcriptional target, the TF RFX6. RA from the lateral plate mesoderm directly activates expression of rfx6 in the posterior foregut endoderm, where it operates at a regulatory crossroad to integrate multiple signaling inputs during gut tube patterning. Specifically, rfx6 first suppresses Wnt-dependent hindgut fate and then inhibits Bmp-dependent fates. Leveraging both Xenopus and human model systems, the authors further dissect Rfx6 downstream mechanism of action, by identifying its target genes and investigating Rfx6-responsive enhancers. Interestingly, they demonstrate that Rfx6 can act as both an activator and repressor in a locus-specific manner.
While the roles of RFX6 in pancreatic, small intestine, and endocrine cell development have been well documented, including in previous studies from this lab., its function in foregut patterning remains less clearly defined. In humans, mutations in RFX6 gene cause the Mitchell-Riley Syndrome, a recessive congenital disorder characterized by variably penetrant, multi-organ defects that reflects its relevance and diverse roles in gut development. This new study provides further insights into the molecular basis of the Mitchell-Riley Syndrome, establishing a framework in which the TF RFX6 integrates RA, Wnt, and Bmp pathways to ensure correct endoderm patterning.
This is an interesting and exceptionally well-executed study, and the results fully support the conclusions drawn by the authors. It provides an elegant demonstration of the power of combining human pluripotent stem cell models with Xenopus embryos to investigate human developmental disorders.

SUGGESTIONS TO AUTHORS
I have only a few comments and questions that the authors may wish to address, either by clarifying specific points in the Results section or by further expanding their Discussion.
1) The extent of conservation in gene regulatory mechanisms and activities between amphibian and humans reported here is noteworthy. It would be also valuable to discuss any species-specific RA targets and TFs the authors might have identified through the integration of the RNASeq datasets. The Venn diagrams in Fig. 1 D and E indicate that only a minority of target genes overlapping between the two species. This raises the question of whether a substantial fraction of RA-responsive targets may be non-conserved.

2) Another interesting observation is the strong similarity between the Xenopus RA and rfx6 LOF transcriptomes, with only a few exceptions. One example is the pancreatic TF Ptf1a, which appears to behave differently. Have the authors followed up on these discrepancies and validated them, for example by assessing Ptf1a expression in Rfx6-Mo or RA-LoF models. Does Ptf1a regulation mediated through an alternative pathway? How do the authors interpret this differential response?

3) There is no discussion here or mention to the role of RA signaling in dorsal-ventral patterning. Previous studies have shown that RA signaling more severely affects the dorsal pancreatic endoderm than the ventral pancreas in both mouse and frog embryos (Chen et al 2004; Molotkov et al 2005; Martin et al. 2005). This asymmetry correlates with the expression pattern of the enzyme Raldh2 (a.k.a., Aldh1a2), which has been reported by others to be enriched in the dorsal splanchnic lateral plate mesoderm in the mouse embryo. Notably, this is consistent with its expression pattern in Xenopus shown here in Figs. 1, 3.

Reviewer 2

SUMMARY OF THE ADVANCE MADE IN THIS PAPER AND ITS POTENTIAL SIGNIFICANCE TO THE FIELD

The Rankin et al. study investigated how Rfx6 integrates upstream gut tube patterning cues with downstream lineage specification programs using human pluripotent stem cell cultures and Xenopus embryos. They observed that RA signaling activates Rfx6 expression in the posterior foregut. Through a series of genetic approaches, they showed that Rfx6 promotes posterior foregut formation by activating posterior foregut lineage regulators (Pdx1 and Onecut1) and Wnt antagonists, while repressing hindgut and anterior foregut lineage regulators (Cdx2 and Nkx2-6) and BMP signaling. They further delineated direct versus indirect targets using cycloheximide (CHX) treatment assay and identified multiple Rfx6-responsive enhancers that support direct regulation of lineage genes. Overall, this is an elegant study that provides solid experimental support for an RA-Rfx6-lineage regulator/Wnt/BMP axis in gut tube patterning. The work adds mechanistic details to how RA interacts with Wnt and BMP pathways to specify organ domains, and the findings are meaningful to the developmental biology community.

© 2026. Published by The Company of Biologists under the terms of the Creative Commons Attribution License (https://creativecommons.org/licenses/by/4.0/).

SUGGESTIONS TO AUTHORS

Below are some comments to further clarify and strengthen the manuscript.
1. The authors reported several effects of RA and Rfx6 at different developmental stages: (1) RA treatment represses pharyngeal TFs at NF 25 (Fig.1). (2) Rfx6 or RA LOF activates Wnt signaling and hindgut TFs at NF25 (Fig.3-4). (3) Rfx6 or RA LOF activates BMP signaling and pharyngeal TFs at NF33 (Fig.3,6). As presented, there are some mismatches between RA treatment and RA LOF phenotypes at NF25, and between RA/Rfx6 LOF phenotypes at NF25 and NF33. Given the time-dependent nature of signaling and lineage TFs during development, it would strengthen the manuscript if the authors could clarify their observations in a clearer temporal scale. For example, does RA/Rfx6 repress competing developmental programs in a stage-specific manner (Wnt/hindgut programs earlier and BMP/pharyngeal programs later)? What happens to the NF25 'hindgut-like' population in Rfx6 LOF by NF33?
2. Regarding the RA-responsive RFX6 enhancers shown in Figure 2, there are additional RFX6 enhancer regions recently identified using CRISPRi (Kaplan et al., 2024, PMID: 39163202). I'd suggest annotating these regions in Figure S2B for comparison. Furthermore, the authors should report the presence or absence of RAR/RXR and other relevant motifs in these regions alongside the human RFX6 intron 14 enhancer. The current motif analysis appears somewhat ad hoc. It would be beneficial to report all motifs identified by CisBP for an unbiased analysis.
3. In Fig.3C-D, the authors showed overlaps in differentially expressed genes (DEGs) between RA LOF and Rfx6 LOF, but there's also a large Rfx6-only (and RA-only) set. It would be informative if the authors could provide some analysis about the condition-specific DEGs. They should also compare the results to those in Figure 1 (with +RA)
4. In Fig.4, the authors showed that Rfx6 LOF upregulates Wnt pathway genes and CDX2 in Xenopus foregut. Previously, Sanchez et al (PMID: 39302049) reported reduced Wnt signaling and reduced CDX2 in RFX6 mutant human iPSC gut tube-stage cultures. Please comment on this discrepancy of Rfx6 function regarding Wnt signaling.
5. The direct repression roles of RFX6 on cdx2 and satb2 etc. is curious. Can the authors provide a mechanistic explanation? RFX6 certainly a DNA binding domain, but it's unclear whether it also has a transcriptional activation and/or repression domain? Does it act through co-factors?

Minor comments:
1. In Fig.2B, part of the foxa1 expression panel is blocked.
2. Typo on page 7 line 175: 'Figs.2C' should be 'Fig.S2C'.
3. Line 201 "dexamethasone (DEX) inducible GR-human RFX6 construct". Please spell out GR the first time this is mentioned.
4. Typo on page 10 line 248: 'a RA-Rfx6' should be 'an RA-Rfx6'.
5. Typo on page 10 line 255: 'Wnt/B-catenin' should be 'Wnt/β-catenin'.
6. Typo in Fig.S3C: 'dowreg' should be 'downreg'.
7. Page 12 line 300, the authors state hoxc10 was indirectly repressed by RFX6, but there is no corresponding figure to support that.
8. The title: "insight into the molecular basis of Mitchell-Riley Syndrome" is quite vague. I'd suggest removing.

First revision

Author response to reviewers' comments

Reviewer 1: SUMMARY OF THE ADVANCE MADE IN THIS PAPER AND ITS POTENTIAL SIGNIFICANCE TO THE FIELD

In the manuscript by Rankin et al., the authors address a long-standing question in developmental biology: how different signaling pathways, such as Retinoic acid (RA), Wnt, and Bmp, and transcription factors (TF) are mechanistically integrated into regulatory networks that control

© 2026. Published by The Company of Biologists under the terms of the Creative Commons Attribution License (https://creativecommons.org/licenses/by/4.0/).

organogenesis. The study focuses on the patterning of the foregut endoderm into distinct organ primordia along the antero-posterior axis. Using a multispecies approach, combining Xenopus embryos and human stem cell models, the authors identified a conserved RA-responsive transcriptional target, the TF RFX6. RA from the lateral plate mesoderm directly activates expression of rfx6 in the posterior foregut endoderm, where it operates at a regulatory crossroad to integrate multiple signaling inputs during gut tube patterning. Specifically, rfx6 first suppresses Wnt-dependent hindgut fate and then inhibits Bmp-dependent fates. Leveraging both Xenopus and human model systems, the authors further dissect Rfx6 downstream mechanism of action, by identifying its target genes and investigating Rfx6-responsive enhancers. Interestingly, they demonstrate that Rfx6 can act as both an activator and repressor in a locus-specific manner. While the roles of RFX6 in pancreatic, small intestine, and endocrine cell development have been well documented, including in previous studies from this lab, its function in foregut patterning remains less clearly defined. In humans, mutations in RFX6 gene cause the Mitchell-Riley Syndrome, a recessive congenital disorder characterized by variably penetrant, multi-organ defects that reflects its relevance and diverse roles in gut development. This new study provides further insights into the molecular basis of the Mitchell-Riley Syndrome, establishing a framework in which the TF RFX6 integrates RA, Wnt, and Bmp pathways to ensure correct endoderm patterning. This is an interesting and exceptionally well-executed study, and the results fully support the conclusions drawn by the authors. It provides an elegant demonstration of the power of combining human pluripotent stem cell models with Xenopus embryos to investigate human developmental disorders.

We thank the reviewer for appreciating the important advances of the study and for the helpful suggestions on how to improve the manuscript.

REVIEWER 1 SUGGESTIONS TO AUTHORS

I have only a few comments and questions that the authors may wish to address, either by clarifying specific points in the Results section or by further expanding their Discussion.

1) The extent of conservation in gene regulatory mechanisms and activities between amphibian and humans reported here is noteworthy. It would be also valuable to discuss any species-specific RA targets and TFs the authors might have identified through the integration of the RNASeq datasets. The Venn diagrams in Fig. 1 D and E indicate that only a minority of target genes overlapping between the two species. This raises the question of whether a substantial fraction of RA-responsive targets may be non-conserved.

Author Response:

While our focus was on the conserved transcriptome downstream of RA, we agree that species-specific targets might be of interest ot some readers. Having said that, we cannot be certain whether the differences are truly species-specific or due to experimental design, and therefore, we prefer not to make too much of this in the paper. **We have added a few sentences about this to the “limitations of the study” section, lines 526-534.**

For the reviewer's benefit, we provide a slightly more extensive explanation here.

a) Some of the apparent species-specific differences are simply different members of the same gene family being RA-regulated, but not reflected in the overlaps (See review Fig.1 below). For example, *hoxc3* and *hoxd4 are* RA-induced in *Xenopus*, while *HOXB3* and *HOXC4* are upregulated by RA in the hPSC-foregut; *IHH* was upregulated in humans, while *dhh* was upregulated in *Xenopus*; *foxi4 was* suppressed by RA in *Xenopus*, while *FOXI3 was* suppressed by RA in humans. Thus, our strict gene symbol matching approach underestimates the “functional” overlap.

b) Some apparent differences might also be due to experimental designs rather than true species-specific differences. One big difference is between *in vivo* tissue, with all its complex cellular interactions prior to isolation, and the simple *in vitro* system. Moreover, we cannot be entirely certain that the stages of *in vivo Xenopus* foregut are the same as *in vitro* human foregut, even though they are roughly equivalent based on key marker genes. Finally, while we used an RA dose

© 2026. Published by The Company of Biologists under the terms of the Creative Commons Attribution License (https://creativecommons.org/licenses/by/4.0/).

known to promote posterior foregut in each system, the concentration and duration of treatment were different due to inherent differences in the systems: 25 nM RA treatment of *Xenopus* foregut endoderm for 16hours (NF15-25) versus 2 μM RA treatment of human PSC foregut endoderm for 24 hours (day 5-6).

c) Of course, some differences are likely to be truly species-specific, but we do not have the data to determine which ones.

Reviewer Figure: potential species-specific differences

NOTE: We have removed unpublished data that had been provided for the referees in confidence.

2) Another interesting observation is the strong similarity between the Xenopus RA and rfx6 LOF transcriptomes, with only a few exceptions. One example is the pancreatic TF Ptf1a, which appears to behave differently. Have the authors followed up on these discrepancies and validated them, for example by assessing Ptf1a expression in Rfx6-Mo or RA-LoF models. Is Ptf1a regulation mediated through an alternative pathway? How do the authors interpret this differential response?

Author Response:

Ptf1a does NOT behave differently. It is a down-regulated gene at NF33 in both the RA LOF and Rfx6 LOF foregut explants, but to different degrees: *ptf1a.S* was log2FC -1.67 in RA LOF and log2FC -4.32 in Rfx6 LOF.

While there is no discrepancy, we have followed up on *ptf1a* expression for the reviewer's benefit using *in situ* hybridization in RA LOF and Rfx6 LOF embryos (see reviewer figure below). Both the dorsal and ventral pancreas domains of *ptf1a* are reduced in both LOF, with the dorsal domain being more sensitive, consistent with the dorsal pancreas being more RA-dependent, as discussed in point-3.

NOTE: We have removed unpublished data that had been provided for the referees in confidence.

3) There is no discussion here or mention to the role of RA signaling in dorsal-ventral patterning. Previous studies have shown that RA signaling more severely affects the dorsal pancreatic endoderm than the ventral pancreas in both mouse and frog embryos (Chen et al 2004; Molotkov et al 2005; Martin et al. 2005). This asymmetry correlates with the expression pattern of the enzyme Raldh2 (a.k.a., Aldh1a2), which has been reported by others to be enriched in the dorsal splanchnic lateral plate mesoderm in the mouse embryo. Notably, this is consistent with its expression pattern in Xenopus shown here in Figs. 1, 3.

Author Response:

In our RA-LOF and Rfx6-LOF, we observed a strong reduction of both dorsal and ventral pancreas gene expression. But agree that published studies on RA have shown a more profound impact on the dorsal than the ventral pancreas, consistent with more robust *aldh1a2* expression in the dorsal late mesoderm. **We have added a few additional sentences citing these papers in the introduction, page 2, lines 62-67:**

"RA also plays roles in dorsal-ventral foregut patterning. The RA-synthesizing enzyme Aldh1a2 is more robustly expressed in dorsal splanchnic mesoderm, and studies have shown that the dorsal pancreatic bud is more sensitive to RA than the ventral bud (Molotkov et al., 2005; Martin et al., 2005; Chen et al., 2004); however, ventral hepatobiliary development does require RA (Guertin et al., 2022; Koike et al., 2019)."

Reviewer 2: SUMMARY OF THE ADVANCE MADE IN THIS PAPER AND ITS POTENTIAL SIGNIFICANCE TO THE FIELD

The Rankin et al. study investigated how Rfx6 integrates upstream gut tube patterning cues with downstream lineage specification programs using human pluripotent stem cell cultures and

© 2026. Published by The Company of Biologists under the terms of the Creative Commons Attribution License (https://creativecommons.org/licenses/by/4.0/).

Xenopus embryos. They observed that RA signaling activates Rfx6 expression in the posterior foregut. Through a series of genetic approaches, they showed that Rfx6 promotes posterior foregut formation by activating posterior foregut lineage regulators (Pdx1 and Onecut1) and Wnt antagonists, while repressing hindgut and anterior foregut lineage regulators (Cdx2 and Nkx2-6) and BMP signaling. They further delineated direct versus indirect targets using cycloheximide (CHX) treatment assay and identified multiple Rfx6-responsive enhancers that support direct regulation of lineage genes. Overall, this is an elegant study that provides solid experimental support for an RA-Rfx6-lineage regulator/Wnt/BMP axis in gut tube patterning. The work adds mechanistic details to how RA interacts with Wnt and BMP pathways to specify organ domains, and the findings are meaningful to the developmental biology community.

We thank the reviewer for appreciating the value of the study and for the constructive suggestions.

SUGGESTIONS TO AUTHORS

Below are some comments to further clarify and strengthen the manuscript.

1a. The authors reported several effects of RA and Rfx6 at different developmental stages: (1) RA treatment represses pharyngeal TFs at NF 25 (Fig.1). (2) Rfx6 or RA LOF activates Wnt signaling and hindgut TFs at NF25 (Fig.3-4). (3) Rfx6 or RA LOF activates BMP signaling and pharyngeal TFs at NF33 (Fig.3,6). As presented, there are some mismatches between RA treatment and RA LOF phenotypes at NF25, and between RA/Rfx6 LOF phenotypes at NF25 and NF33. Given the time-dependent nature of signaling and lineage TFs during development, it would strengthen the manuscript if the authors could clarify their observations in a clearer temporal scale. For example, does RA/Rfx6 repress competing developmental programs in a stage-specific manner (Wnt/hindgut programs earlier and BMP/pharyngeal programs later)?

Author Response:

We agree that the temporal dynamics warrant a better explanation. Our transcriptomic data indicate that RA suppresses both hindgut and pharyngeal markers at both NF25 and NF33. Rxf6, on the other hand, only suppresses Wnt-dependent hindgut markers at NF25, and it is not until NF33 that it suppresses both hindgut and BMP-dependent pharyngeal markers. This was shown in the original GSEA in Fig. 3I, but we obviously did not make that clear. We conclude that RA and Rfx6 are both required in the posterior foregut to suppress alternative hindgut and pharyngeal programs, with RA activating first, consistent with it being upstream of Rfx6. Then Rfx6 reinforces the suppression of pharyngeal fates while continuing to suppress hindgut fates. We have clarified this in the **results and discussion on lines 232-235, 261-266, 277-280, and 451-453**.

1b. What happens to the NF25 'hindgut- like' population in Rfx6 LOF by NF33?

Author response:

NOTE: We have removed unpublished data that had been provided for the referees in confidence.

As mentioned above at NF33, HG TFs (*cdx2, satb2, ventx2*) and posterior hox factors (*hoxa9/b9/c9*, *hoxc10/d10, hoxd11*) remain up-regulated in both the Rfx6 LOF and RA LOF foregut explants. This was shown in the original GSEA in Fig. 3I and in the supplementary DEG lists, but again, that obviously wasn't clear**. We now state this in the text, lines 232-235 and 261-266, and indicate some key hindgut genes in the Fig. 3G heatmap, as well as include an additional heatmap in Supplemental Fig. S3J (shown here to right)**

2a. Regarding the RA-responsive RFX6 enhancers shown in Figure 2, there are additional RFX6 enhancer regions recently identified using CRISPRi (Kaplan et al., 2024, PMID: 39163202). I'd suggest annotating these regions in Figure S2B for comparison. Furthermore, the authors should report the presence or absence of RAR/RXR and other relevant motifs are present in these regions, alongside the human RFX6 intron 14 enhancer.

Author Response:

© 2026. Published by The Company of Biologists under the terms of the Creative Commons Attribution License (https://creativecommons.org/licenses/by/4.0/).

Thank you for pointing out the Kaplan et al 2024 study. We agree it is relevant, and we now cite this **paper and have added a sentence in the results section on lines 164-166:**

"The DNA cis-regulatory elements governing RA-dependent *RFX6* expression in the embryonic gut tube have not been characterized, although recent studies have identified some enhancers that may regulate *RFX6* during pancreas development (Kaplan et al., 2024)."

However, we have chosen not to annotate the Kaplan et al 2024 putative *RFX6* enhancers in our Figure S2, since these are NOT bound by RXR in the hPSC-foregut tissue (see reviewer Figure below). RA responsiveness was an essential criterion for examining putative *RFX6* CREs in our study. We felt that annotating those regions in our figures would distract from the main point.

Reviewer Figure Kaplan et al 2024 putative *RFX6* enhancers highlighted in green compared to the RXR-bound intron 14 enhancer highlighted in yellow.

NOTE: We have removed unpublished data that had been provided for the referees in confidence.

2b. The current motif analysis appears somewhat ad hoc. It would be beneficial to report all motifs identified by CisBP for an unbiased analysis.

An unbiased CisBP or Jaspar analysis of the 699 bp RFX6 intron 14 enhancers identified >1500 putative TF DNA-binding motifs (90% confidence), most of which are probably not biologically relevant. We don't think there is value in showing this list; readers can perform their own analysis. Having said that, we do take the reviewer's point. Therefore, in addition to the RXR/RAR motifs, we now also report the predicted FOXA and GATA motifs, as well as the RXR/RAR motifs, since Lee et al. (2019) and Vinckier et al. (2020) indicate that these are likely critical in the posterior foregut and pancreas. **See new Table S2, and lines 181-182)**.

3. In Fig.3C-D, the authors showed overlaps in differentially expressed genes (DEGs) between RA LOF and Rfx6 LOF, but there's also a large Rfx6-only (and RA-only) set. It would be informative if the authors could provide **(3a)** some analysis about the condition-specific DEGs. They should also **(3b)** compare the results to those in Figure 1 (with +RA).

Author Response:

While our goal was to identify the conserved genes co-regulated by RA and Rfx6, we agree that some readers could be interested in learning more about the other condition-specific DEGs. We had planned on including such analyses in the original manuscript, but did not due to space limitations. If the Development editors allow it, we will add additional figures and text described below. We broke down our answer to 3a and 3b as indicated above.

3a: condition-specific DEGs: We propose to add a **new supplemental figure, Supplemental Figure S4 (shown below),** indicating TFs uniquely regulated at each time (NF25, NF33) in each condition (RA LOF or Rfx6 LOF), as well as GO analyses of the gene sets uniquely regulated by RA and Rfx6. **We have added new text on lines 258-263.**

Some of the main findings are:

i) Some of the apparent differences in RA versus Rfx6 specific target genes were due to temporal dynamics as described above for point-1. For example, at NF25, a subset of aFG/pharyngeal TFs *(foxq1, irx2, nkx2-3, nkx2-5, nkx2-6, otx1, otx2, pax1, pax9)*, and a subset of posterior Hox factors *(hoxd8, hoxb9, hoxd9, hoxa10, hoxa11, hoxc12, hoxc13)*, were already up-regulated in RA LOF as compared to Rfx6 LOF. It was not until NF33 that these were upregulated in the Rxf6 LOF, consistent with RA acting upstream of Rfx6. In addition, the well-known direct RA target *hnf1b* was down-regulated at NF25 in RA LOF, but it was not significantly down-regulated in the Rfx6 LOF until NF33.

© 2026. Published by The Company of Biologists under the terms of the Creative Commons Attribution License (https://creativecommons.org/licenses/by/4.0/).

ii) There are clearly some DEGs that are specific to RA LOF and RFX6 LOF. In the new supplemental Fig. S9, we now list some of these and show the GO term enrichment for these groups. Interestingly, although RA and Rfx6 regulate distinct genes, they tend to show similar GO enrichment, including cell migration, ECM organization, and mesoderm lineage development. We have chosen not to comment extensively on this in the text, as it detracts from the main point, but the data is now available for exploration.

NOTE: We have removed unpublished data that had been provided for the referees in confidence.

3b. Compare the Fig. 3 results to those in Figure 1 (with +RA).

Author response:

We agree that comparing RA (GOF) activated genes (Fig.1) to those that require RA or Rfx6 (LOF from Fig.3) is valuable.

We had debated whether to include such an analysis in the original manuscript, but did not due to space limitations. If the editors agree, we will now include an **additional supplementary Fig. S5** showing this comparison of "Figure 1 genes to Figure 3 genes", and additional table S5 with the corresponding gene lists.

In the **new supplemental figure S5 analysis and table S5**, we combined the two LOF time points (NF25+33) for each LOF and compared them with the NF25 RA GOF. This analysis identified a set of genes for which RA was necessary and sufficient for their expression, including *rfx6*, along with pFG TFs *gata4, hnf1b, pdx1, osr1, onecut1*, and *hoxa/b/c/d 1-5*. On the other hand, many genes suppressed by RA GOF were indeed upregulated in the RA LOF and/or RFX6 LOF, such as pharyngeal genes. This analysis also identified some genes in LOF conditions (like Wnt ligands) that were mesodermal or non-cell-autonomously regulated through interactions with the mesoderm, which was absent in the RA GOF experiments in Fig. 1. Given the word limit constraints and to avoid detracting from the paper's main focus, we have kept our discussion of these observations to a minimum on **lines 265-273**, but the data are available for readers to explore if they wish.

NOTE: We have removed unpublished data that had been provided for the referees in confidence.

4. In Fig.4, the authors showed that Rfx6 LOF upregulates Wnt pathway genes and CDX2 in Xenopus foregut. Previously, Sanchez et al (PMID: 39302049) reported reduced Wnt signaling and reduced CDX2 in RFX6 mutant human iPSC gut tube-stage cultures. Please comment on this discrepancy of Rfx6 function regarding Wnt signaling.

Author response:

We acknowledge that the differences in Rfx6 function between the posterior foregut and the midgut (small intestine) are a conundrum. But to be honest, we don't fully understand the mechanisms.
We are hesitant to speculate in the paper without more data. However, for the reviewer's benefit, below we outline several possibilities that are not mutually exclusive.

Our interpretation is that the difference is due to context-dependent regulation of RFX6 by Wnt signaling and *CDX2* expression in the distinct cellular contexts of the foregut and midgut.

1. RFX proteins form homodimers or heterodimers that can bind different co-factors to act as activators or repressors (as stated in the original discussion). RFX6 may interact with different protein partners in the foregut versus the small intestine, which could result in context- dependent transcriptional outcomes on *CDX2* and/or Wnt signaling.
2. Another complication is combinatorial TF regulation of enhancers. The foregut is SOX2+/CDX2- while the midgut is SOX2-/CDX2+. This cell state is set very early, prior to initial RFX6 expression, in part through complex combinatorial TF feedback loops that likely influence how RFX6 acts on the CDX2 enhancer in the foregut versus the midgut.

© 2026. Published by The Company of Biologists under the terms of the Creative Commons Attribution License (https://creativecommons.org/licenses/by/4.0/).

3. Finally, in the foregut, Rxf6 acts in part by regulating Sfrps to modulate paracrine Wnt signaling from the mesoderm. In Sanchez et al. (2024), we assayed the small intestine and duodenum, which do not normally express Sfrps, so this component was absent from the in vitro system.
4. Thus, the differences in WNT/RFX6-modulated *CDX2* expression could be due to differences in GI tissues, tissue-specific TFs, RFX6-interacting cofactors/or the interaction with the mesoderm, which vary between in vivo- and in vitro-generated organoids. We plan to examine this fascinating and important question in the future.

5. The direct repression roles of RFX6 on cdx2 and satb2 etc. is curious. Can the authors provide a mechanistic explanation? RFX6 certainly a DNA-binding domain, but it's unclear whether it also has a transcriptional activation and/or repression domain? Does it act through co-factors?

As we explained in the discussion (lines 482-488), it is well known that RFX TFs can act as both activators and repressors depending on the interacting heterodimer partners, other RFX family members, and other co-factors. The fact that Rfx6 can act as both an activator and a repressor on different enhancers in the same tissue is a novel observation in our study. We agree that this is fascinating, and we plan to explore it in the future.

Minor comments:

Thank you for taking the time to point these out to us. We appreciate it and have corrected them.
1. In Fig.2B, part of the foxa1 expression panel is blocked. Corrected.
2. Typo on page 7 line 175: 'Figs.2C' should be 'Fig.S2C'. Corrected.
3. Line 201 "dexamethasone (DEX) inducible GR-human RFX6 construct". Please spell out GR the first time this is mentioned. We have included this text: “Kolm and Sive, 1995; GR refers to the ligand binding domain of the human glucocorticoid receptor which also binds and is activated by DEX).
4. Typo on page 10 line 248: '**a** RA-Rfx6' should be '**an** RA-Rfx6'. Corrected.
5. Typo on page 10 line 255: 'Wnt/**B**-catenin' should be 'Wnt/**β**-catenin'. Corrected.
6. Typo in Fig.S3C: 'dowreg' should be 'downreg'. Corrected.
7. Page 12 line 300, the authors state hoxc10 was indirectly repressed by RFX6, but there is no corresponding figure to support that.

We apologize for the oversight. This RT-qPCR data was in a previous version of supplemental Fig.S5 and has now been re-inserted/ included back into the supplemental figure, and with the new numbering / additional supplements, this is **now supplemental Fig.S7.**

8. The title: "insight into the molecular basis of Mitchell-Riley Syndrome" is quite vague. I'd suggest removing. Unless the editors disagree with us, we prefer to keep it, since recessive *RFX6* mutations cause Mitchell-Riley Syndrome. The networks regulated by RFX6 in early development are poorly understood, and our data provide insight into early RFX6-dependent networks that will almost certainly be disrupted in Mitchell-Riley Syndrome patients.

Second decision letter

MS ID#: dev.205552R1

MS TITLE: A Retinoic Acid-Rfx6 cascade modulates Wnt/Bmp-dependent gut tube patterning: insight into the molecular basis of Mitchell-Riley Syndrome

AUTHORS: Scott A. Rankin, Jacek Biesiada, Kyle E. McCracken, Riya K. Patel, Mehek Rana, David M. Luedeke, Daniel O. Kechele, James M. Wells and Aaron M. Zorn

Dear Aaron,

© 2026. Published by The Company of Biologists under the terms of the Creative Commons Attribution License (https://creativecommons.org/licenses/by/4.0/).

I am happy to tell you that your manuscript has been accepted for publication in Development, pending our standard publication integrity checks.

Reviewer 1

SUMMARY OF THE ADVANCE MADE IN THIS PAPER AND ITS POTENTIAL SIGNIFICANCE TO THE FIELD

The authors have addressed all my concerns and I believe that the current version has improved remarkably. Therefore, I recommend the manuscript to be accepted.

Reviewer 2

SUMMARY OF THE ADVANCE MADE IN THIS PAPER AND ITS POTENTIAL SIGNIFICANCE TO THE FIELD

The paper identified the Retinoic Acid-Rfx6 cascade and its role in gut tube patterning.

SUGGESTIONS TO AUTHORS

The authors have addressed all my concerns. No further comments.

© 2026. Published by The Company of Biologists under the terms of the Creative Commons Attribution License (https://creativecommons.org/licenses/by/4.0/).
